# Microbial rhizosphere and topsoil communities in young reforestation plantations

**DOI:** 10.3389/fmicb.2026.1915742

**Published:** 2026-08-26

**Authors:** Jenny Vivian, Alison Shapcott, Robin L. Chazdon, David J. Lee

**Affiliations:** 1 Forest Research Institute, University of the Sunshine Coast, Sippy Downs, QLD, Australia; 2 Centre for Bioinnovation, University of the Sunshine Coast, Sippy Downs, QLD, Australia; 3 School of Science, Technology and Engineering, University of the Sunshine Coast, Sippy Downs, QLD, Australia

**Keywords:** *Acacia mangium*, bacteria, fungi, rhizosphere, soil, soil microbes, tropical monocultures

## Abstract

Relationships between microbial communities of the rhizosphere and plants can affect the successful establishment of plantations and their modification for forest restoration efforts. Recognising these fungal and bacterial assemblages is important for *Acacia mangium* plantations, as they are widely established in reforestation programs across the tropics. However, the microbial community associated with the rhizosphere of *A. mangium* and of plants often thriving within or in the proximity of *A. mangium* plantations is understudied. We hypothesise that microbial assemblages of the topsoil, hosted in the rhizosphere of the planted species and of the co-dominant plant species in the landscape, may differ. In the examined 2-year-old plantation, the plant community is dominated by the eudicotyledonous *A. mangium* and by monocotyledonous species (*Cocos nucifera, Imperata cylindrica,* and *Saccharum* spp.), whereas in the 10-year-old plantation, eudicotyledonous trees (*Swietenia macrophylla,* and *Pterocarpus indicus*) dominate the area. Given the diverse abundance of plants of different phylogenetic traits, we hypothesised a difference in the topsoil microbial community based between these two evolutionarily distinct Angiosperm lineages. Through high-throughput sequencing, we identified the taxonomic and putative functional traits of fungal and bacterial taxa in the rhizospheres of the cultivated and co-dominant plant species. Disproving our hypothesis, we found comparable taxonomic and functional community composition of fungi and bacteria among the rhizospheres tested and between these and the topsoil of the same plantation, without significant differences among mono- and eudicotyledonous species. Still, *C. nucifera* seemed to display a markedly unique rhizosphere community, and ectomycorrhizal fungi were abundant in the 2- and 10-year-old *A. mangium* plantings. Results support the role of topsoil as a reservoir for the microbial community, on which plants act by filtering the taxa hosted in their rhizosphere. In our context, a non-significant difference in the microbial composition may have reflected comparable ecological adaptations and needs, but further research is suggested, given the small sample size of this study. Our research provides insights into the microbial assemblages of the rhizospheres and topsoil of *A. mangium* plantations in a region of the Philippines, helping to disentangle the relationship between the soil microorganisms and plant rhizospheres during forest restoration.

## 1 Introduction

The analysis of microbial communities of the rhizosphere allows understanding which plant species are associated with the presence of specific fungi and bacteria. Soil microbes play a fundamental role in carbon sequestration and nutrient acquisition, among other ecosystem processes. For instance, they can improve carbon sequestration through their biomass (Lemke and DeSalle, 2023; McCulloch et al., 2024; Zhang et al., 2025) or participating in the balance of nutrient cycles (e.g., through sulfate respiration; Demin et al., 2024). The variation of the tree community is commonly explored in forest restoration, but changes in the composition of soil fungi and bacteria associated with the rhizospheres of the dominant plant species are rarely studied in this context.

Reforestation projects have been widely implemented in the tropics to counteract the effects of land degradation and climate change (Griscom et al., 2017). In this context, monocultures are often established (Liu et al., 2018) with the purpose of improving the livelihood of local communities and rapidly regaining tree cover in degraded areas. The need for a fast-growing and economically valuable tree made *Acacia mangium* one of the leading tree species being planted (Potter et al., 2006), given its capabilities of thriving in harsh conditions (Krisnawati et al., 2011; Hegde et al., 2013). Such plantations were generally left unmanaged within a few years of their establishment, often due to socio-economic reasons (Chokkalingam et al., 2006; Gregorio et al., 2015). Subsequently, no assessments of their environmental modifications were conducted over time. In some tropical areas, plantations of *A. mangium* resulted in the uncontrolled spread of this species (e.g., in Malaysia; Datiles and Acevedo-Rodriguez, 2022). However, in the Philippines, the cessation of management of *A. mangium* plantations and occasional harvesting favoured the ingression of native vegetation (Vivian et al., 2026a). Identifying the soil microbes associated with the rhizosphere of the planted species in this area would enhance the understanding of how these widespread monocultures, originally intended for reforestation, influence recovery of soils and ecosystem processes.

Analysing the rhizosphere of co-dominant plants can enhance the understanding of the role these species play in the recovery of ecosystem services, given their ecological role deriving from hosting specific microbial assemblages among their roots. In a related study, fungal and bacterial soil communities of the bulk topsoil (hereafter referred to as “topsoil”) were analysed in a matrix of naturally regenerating *A. mangium* plantations of different ages in the Philippines (Vivian et al., 2026b). However, the microbial assemblages of the rhizosphere of the other most abundant plant species were not considered. Numerous studies demonstrate the growth improvement of *A. mangium* in plantations following inoculation of mycorrhizal fungi (e.g., Aggangan et al., 2010; Dela Cruz et al., 1988) and nitrogen-fixing bacteria (e.g., Galiana et al., 1998; Sethi et al., 2021). Additionally, some evidence of distinct fungal assemblages highlights the association of *A. mangium* with ecto- and endomycorrhiza (Aggangan et al., 2010), which are mainly part of the Basidiomycota, Ascomycota, and Glomeromycota phyla, respectively (Rosendahl, 2008; Tedersoo et al., 2010). The bacterial community in the rhizosphere of this tree species is usually dominated by Pseudomonadota (formerly called Proteobacteria), Acidobacteria, and Actinobacteria (Yuan et al., 2022). Still, a knowledge gap remains regarding the microbial community of its rhizosphere under field conditions (except a single case in Brazil, see Pereira et al., 2017). Such a lack of knowledge is more evident where other plant species dominate the area, potentially influencing the composition of the microbial community.

Previous studies explored the association of microbial taxa with the rhizosphere of dominant plants in the tropical landscape. Still, this knowledge is limited in *A. mangium* plantations, especially in the Philippines. Thus, we implemented an analysis of microbial communities in 2- and 10-year-old monocultures of this species in this country. Specifically, we considered *A. mangium* plantations of different ages to capture the variation of the microbial community in the rhizosphere of this species and in those of five other dominant plants present in the areas. The co-dominant plant species differ between the ages of the plantation, given the natural regeneration occurring in them (Vivian et al., 2026a). Thus, we explored the fungal and bacterial community composition of the rhizosphere of *A. mangium* (Fabaceae; the main planted tree species), and of the five other most common plant species, to explore their influence on the topsoil microbial composition compared to that of *A. mangium*: *Swietenia macrophylla* (Meliaceae; exotic species occasionally planted within the 10-year-old plantation, known as “Mahogany”), *Pterocarpus indicus* (Fabaceae; native species, planted to enrich the 10-year-old plantation, locally called “Narra”), *Cocos nucifera* (Arecaceae; coconut palm, commonly present in the 2-year-old plantation, probably deriving from nearby plantations), *Imperata cylindrica* (Poaceae; “Cogon grass,” dominating the landscape of the 2-year-old plantation), and *Saccharum* spp. (Poaceae; “sugarcane,” also present in the 2-year-old plantation). We compared the taxonomic and functional profile of the microbial assemblages between the rhizospheres and with the topsoil hosting the analysed plants.

Phylogenetically conserved traits of these dominant plant species could be a source of differentiation for their microbial communities. Previous studies highlighted a difference in the fungal and bacterial communities of the rhizosphere between eudicotyledonous and monocotyledonous plants (hereafter referred to as “eudicots” and “monocots”), mainly due to their root exudates, structure, and collaboration with specific microbial taxa for nutrient acquisition strategies (Badri et al., 2009; Dennis et al., 2010; Lakshmanan et al., 2012; Carvalhais et al., 2013; Lebeis et al., 2015; Zhou et al., 2025). In this study, *A. mangium*, *S. macrophylla*, and *P. indicus* belong to the eudicot plants (The Angiosperm Phylogeny Group et al., 2016), while *C. nucifera, I. cylindrica, and Saccharum* spp. to the monocots (Simpson, 2010). The divergence of the monocots and eudicots lineages occurred around 150 million years ago (Zuntini et al., 2024). However, the effect of the phylogenetic traits over that of the species-specific ecological niche and requirements remains understudied, as the similarity of the taxonomic and functional traits of the microbial community belonging to the rhizospheres of phylogenetically distant plants (Yu and Hochholdinger, 2018).

Previous analyses indicate the potential abundance of specific taxa in the examined rhizospheres. A recent study on the microbial communities of *C. nucifera*, conducted on Yongxing Island (China), reported the dominance of Ascomycota and Pseudomonadota (Bao et al., 2020). The rhizosphere of commercial *Saccharum* spp. has also been extensively investigated (mainly in laboratory or monocultures), revealing an abundance of Ascomycota and Basidiomycota phyla for fungi (e.g., in Brazilian sugarcane fields, Moneda et al., 2022), and Pseudomonadota, Actinobacteria, Candidatus *Saccharibacteria*, Acidobacteria, Bacillota (also known as “Firmicutes”), Gemmatimonadetes, and Bacteroidetes bacteria phyla (Dong et al., 2018; Moneda et al., 2022). The microbial assemblages of the rhizospheres of the other three plant species considered (*I. cylindrica*, *S. macrophylla*, and *P. indicus*) are poorly studied (but see Bonner et al., 2019 for analysis in *S. macrophylla* plantation).

From past analyses undertaken in other ecosystems, we expected a high relative abundance of endomycorrhizal fungi (i.e., Glomeromycota phylum; Rosendahl, 2008), specifically arbuscular mycorrhiza, in the rhizosphere of eudicots and potentially in the nearby topsoil where they occur (Weishampel and Bedford, 2006; Albarracín Orio et al., 2016). Given the traits of oligotrophic bacteria (Dragone et al., 2024) that thrive in harsh conditions (e.g., high temperature, low humidity, nutrient-poor), we anticipated a higher relative abundance of Bacillota, Chloroflexota, Actinomycetota, Planctomycetota, and Pseudomonadota associated with rhizospheres of the monocots, further corroborated by their different root exudates compared to eudicots (McLaughlin et al., 2023). Overall, we hypothesized: (i) a statistically significant difference in microbial community composition between eudicots and monocots, but not between species belonging to the same group, for a stronger effect of phylogenetic traits over ecological niches and requirements; (ii) a higher relative abundance of symbiotrophic fungi in all eudicot species (especially for *A. mangium*) compared to monocots. We also hypothesized (iii) a greater relative abundance of ectomycorrhizal fungi in older *A. mangium* trees, due to the development of symbiotic relationships over time, and (iv) a dominance of oligotrophic bacteria taxa in the monocots plant group, including the phyla known to be present in the *Saccharum* spp. rhizosphere reported above. Therefore, we aimed to answer the following questions: (i) How do microbial richness and community composition differ among the rhizospheres of *A. mangium* (at two stand ages), and those of the other dominant plant species? (ii) Do the microbial assemblages differ between rhizospheres and topsoils? (iii) Do eudicots and monocots differ in their rhizosphere microbial community composition? (iv) Do the putative functions of the microbial communities of the rhizospheres and topsoil differ among species and, for *A. mangium*¸ over time? Are there presumed microbial functions, particularly important for tropical forest restoration and carbon sequestration, whose relative abundance is notably high in the rhizosphere of certain species compared to others? By exploring the microbial assemblages of the rhizospheres of dominant plants in *A. mangium* monocultures of the Philippines, this study establishes the baseline for further investigations to unravel potential associations between plants, topsoil, and microbes. Indeed, our work was constrained by the limited number of samples and plantation ages; thus, we recognise the limitation in unravelling long-term associations between microbial assemblages and *A. mangium* trees (and other dominant plant species). Hence, we suggest future research should focus on the rhizosphere of this species in older plantations and controlled laboratory settings. Such research avenues can potentially recognise the influence of relationships between plants and microbial taxa on ecosystem functioning and services. For instance, the analyses may unravel a high abundance of topsoil chemicals given the large presence of the plants to which the bacterial taxa synthesising such compounds are associated.

## 2 Methods

### 2.1 Study site and sampling

In September 2024, samples were gathered in the study site located on the tropical island of Biliran, Philippines (Supplementary Figure S1). Here, the climate is tropical without marked seasonal variations (Nguyen et al., 2012), and the native vegetation is evergreen tropical rainforest, over homogeneous basaltic deposits (Peque and Hölscher, 2014; Mendoza et al., 2019). On the island of Biliran, numerous *A. mangium* plantations are present, of different ages and stages of natural regeneration. Due to limited budget and time, our research focused on the rhizospheres of dominant plants from 2- and 10-year-old *A. mangium* plantations. Aboveground aspects of the system and topsoil fertility of the same locations showed that such a timeframe was sufficient to detect significant environmental changes (Vivian et al., 2026a). Extensive descriptions of methods and of the study area location are reported in (Vivian et al., 2026a, b).

Samples of the rhizosphere were collected for three individuals of each species (six for *A. mangium,* three for each plantation age) in the 2- and 10-year-old *A. mangium* plantations, by scrapping at least three times the soil attached to the roots of selected plants in the topsoil (0–10 cm), to obtain approximately 1 g of soil. The roots were manually uncovered by removing the litter layer, where present, avoiding contamination by wearing sterile gloves. The roots included in the study were located near the trunk and stem for woody and herbaceous plants, respectively. Thus, we collected the soil samples in the zone defined more precisely as “ectorhizosphere,” which “consists of soil immediately adjacent to the root” (Prashar et al., 2014). The soil was collected in replicates and stored in sterile vials, containing 1 mL of DNA/RNA Shield (Zymo Research, USA) buffer for DNA preservation. We ensured that the sampled roots belonged to the selected species by tracing them to the trunk (or stem) of the identified plant.

**Figure 1:**
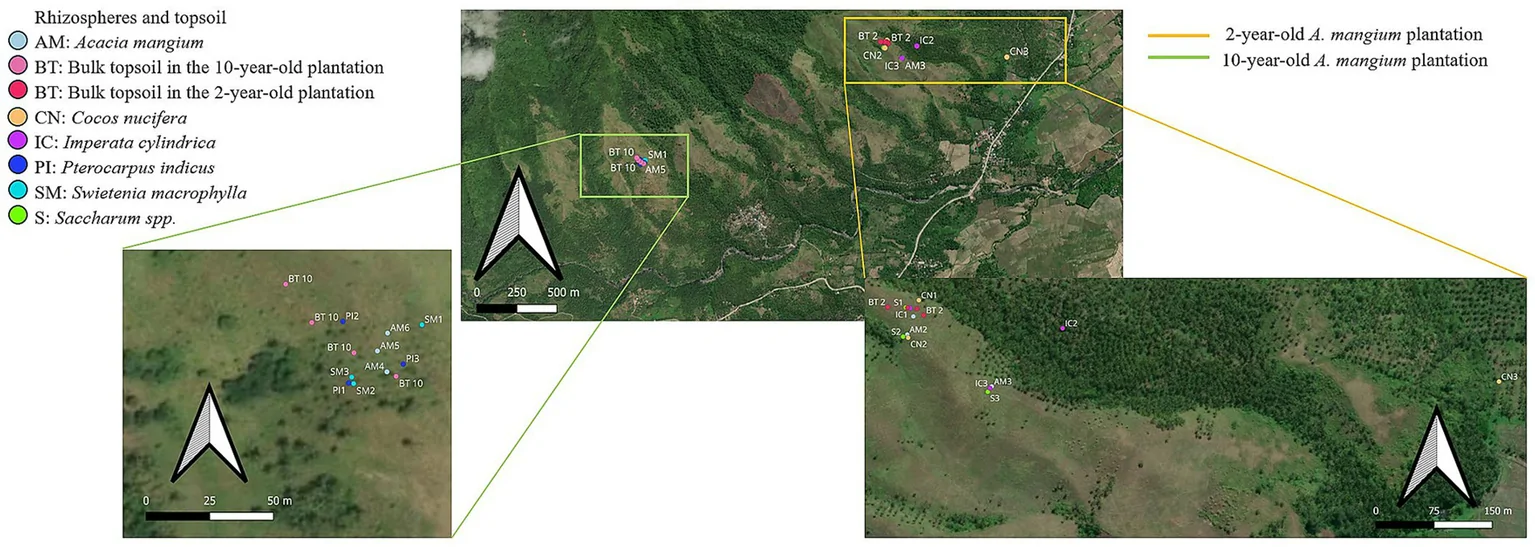
Location of the sampled rhizospheres (*n* = 3 for each plant species in each plantation) and topsoil (*n* = 4 for each plantation) collected in 2-, and 10-year-old *Acacia mangium* plantations in Biliran, Philippines.

In the 2-year-old plantation, *A. mangium*, *C. nucifera*, *Saccharum* spp., and *I. cylindrica* dominated the landscape; thus, we sampled the rhizospheres of three individuals of each of these species (selecting larger plants when possible). In the 10-year-old plantation (established in 2014), we sampled three distinct rhizospheres of the cultivated *A. mangium*, and planted *S. macrophylla* and *P. indicus* trees, which were established as an enrichment planting in the *A. mangium* plantation at age four (i.e., 2018–2019). No grass species were sampled in the 10-year-old plantation due to their scarcity. All selected plants were located at least 3 to 5 m apart (Figure 1). The topsoil (0–10 cm) samples considered for comparisons were collected as part of a related study (Vivian et al., 2026b). The samples considered in this study consisted of a total of four (bulk) topsoil samples from the 2-year-old and four from the 10-year-old plantations, which were originally part of a larger experimental design consisting of four transects for each plantation (and other sites not considered in this study). Specifically, topsoil samples were collected between one and two weeks before the collection of the rhizosphere samples, at the centre of four 5 × 5 m plots, spaced 10 m apart, and organized in one transect disposed over the altitudinal range (Supplementary Figure S2). In our research, we included only the transect of plots located in the proximity of the sampled trees (within a distance between 3 and 40 m from the transects, according to the plants availability) to improve the comparability of the results. The rhizosphere samples were gathered outside the plots in which the topsoil was sampled due to the lack of the selected species within these plots, to ensure spatial independence between the rhizosphere samples collected, and to prevent the collection of contaminated samples, as the transects were established before the collection of the rhizosphere samples, but after that of the topsoil. All samples were treated separately as replicates. The protocol adopted included field control samples; thus, vials were handled similarly to those in which the topsoil was inserted, but retained empty of this substrate (i.e., only containing the DNA/RNA Shield buffer). We carefully discuss the results of the analysis, acknowledging the limitations of our study and recognising that, ideally, the experimental design should consider the gathering of topsoil and rhizosphere samples sequentially and in the same location, for a more robust comparison of the results. Still, the selection of the plants in the proximity of the plots and spaced from each other, and the disposition of the transects within the different plantations made them relatively ideal representatives of the rhizospheres and topsoil communities in our context of study and within the constraints of our research.

To provide better contextualization of the assessed microbial community, data on the height and diameter at breast height (DBH) were collected for each of the selected trees (Table 1). Additional topsoil abiotic parameters, air temperature, and humidity were recorded in the location of the topsoil samples and reported in Vivian et al. (2026a) and Vivian et al. (2026b). While not perfectly matched to the samples of the rhizosphere, the proximity of soil abiotic parameters measurements is well within the documented decay distance of 200 m (Vivian et al., 2026a) Table 1.

**Table 1:** Dimensional traits of the vascular plants associated with the analysed rhizospheres.

Plant species	DBH (cm)	Height (m)	Location
*Acacia mangium*	8.9	6.1	2-year-old plantation
	3.5	3.1	
	2.2	2.5	
*Acacia mangium*	38.6	32.2	10-year-old plantation
	0.7	1.5	
	30.9	18.6	
*Cocos nucifera*	25.2	11	2-year-old plantation
	31.3	11.4	
	28	11.5	
*Swietenia macrophylla*	8.6	8.3	10-year-old plantation
	4^‡^	6^‡^	
	4	7.8	
*Pterocarpus indicus*	<0.5	0.9	
	0.5	1.8	
	0.5	1.75	

The “^‡^” indicates the dimension of the *S. macrophylla* tree whose sample was discarded due to low sequencing depth.

### 2.2 Environmental DNA extraction and sequence processing

Rhizosphere and topsoil samples were processed similarly and simultaneously. The replicates collected were analysed at Sequench Ltd. (Nelson, Aotearoa, New Zealand) between November and December 2024. For fungal identification, the internal transcribed spacer 2 (ITS2) region of the nuclear ribosomal RNA (rRNA) gene was studied (White et al., 1990), while the V3-V4 region of the nuclear small-subunit ribosomal RNA (16S rRNA) gene was used for the recognition of the present bacteria (Klindworth et al., 2013). Considering established guidelines (Kozich et al., 2013), the primers used (with Illumina overhang adaptors) were: ITS3_Illumina_tag 5′-GCATCGATGAAGAACGCAGC-3′ for forward transcription, and ITS4_Illumina_tag 5′-TCCTCCGCTTATTGATATGC-3′ for reverse transcription, for fungi. For bacteria, the NexR-16SFWD 5′-CCTACGGGNGGCWGCAG-3′, and the NexR-16SREV 5′-GACTACHVGGGTATCTAATCC-3′ were the primers used for forward and reverse transcription, respectively. Details on the amplification of the target genes, purification, and normalisation steps are thoroughly reported in Vivian et al. (2026b). Amplicons were then cleaned and indexed (see Vivian et al., 2026b for details). Afterwards, high-throughput sequencing was conducted on the Illumina NextSeq 2000 platform with the NextSeq Reagent X-LEAP kit P1-600 cycle (2 × 301 bp) (Illumina, San Diego, CA, USA), with the library pool being diluted to 600 pM and a 25% PhiX spike.

Cleaning, truncation of the reads, filtering, dereplication, and denoising steps of the sequences were conducted as described in Vivian et al. (2026b), ensuring the presence of bacterial and fungal taxa in the dataset. The “DADA2” package (Callahan et al., 2016), ‘assignTaxonomy’ script, and the RDP classifier (Wang et al., 2007), with default settings, allowed for the fungi taxonomic assignment given the UNITE database (version 10.0) (Abarenkov et al., 2024). For bacteria, the taxonomic assignment was conducted using the SILVA database (version 138.2) (Quast et al., 2013). Possible functions expressed by the detected taxa were identified using the “microeco” package v1.10.0 (Liu et al., 2021), the FUNGuild database (Nguyen et al., 2016) for fungi, and the FAPROTAX database v1.2.10 (Louca et al., 2016) for bacteria. All analyses were conducted in the R environment (R Core Team, 2024). More details on the bioinformatic pipeline are reported in Vivian et al. (2026b).

Rarefaction curves were created for both fungi and bacteria, enabling the identification of a threshold for the control of the sampling effort, balancing it with the taxonomic coverage (Schloss, 2024). Specifically, this step allows the determination of adequate sequencing depth and the standardisation of the read number across samples (Supplementary Figures S3, S4 for rhizosphere samples). This step was conducted with the “vegan” v2.6-8 (Oksanen et al., 2025) and “ggplot2” v3.5.1 (Wickham, 2009) packages in the R environment (R Core Team, 2024). A different threshold was selected for the rhizosphere and topsoil samples to minimise information loss, ensuring that the biological comparisons are based on equivalent levels of community saturation. For instance, topsoil samples reached the plateau, indicating an adequate sequencing depth before those of the rhizosphere. Selecting a threshold for an even sequencing depth based on the topsoil samples would have led to the loss of information about the ASVs present in the rhizosphere samples. Conversely, choosing a threshold adequate only for the rhizosphere samples would have required discarding multiple topsoil samples. For the communities of the assessed rhizospheres, data were rarefied to 33,404 reads for fungi, resulting in one sample being discarded (from the *S. macrophylla* rhizosphere, coded “SM2” in Figure 1). Bacterial sequences were rarefied to 30,470 reads, with no samples being removed after this step. For topsoil samples, fungi sequences were rarefied to 63,341, and bacteria to 38,483 reads. In this case, none of the samples gathered and considered for this study were discarded after this process. Importantly, in Supplementary material (Section 3) we report the results of using the same threshold for all samples (the most stringent one, thus 33,404 for fungi and 30,470 for bacteria), showing that the results are consistent with those obtained with differential (but more defined for each case) scores. Raw sequence reads on the topsoil were deposited in the NCBI Sequence Read Archive under the BioProject ID and accession number PRJNA1295595, while raw reads of the rhizosphere can be accessed through PRJNA1302026.

### 2.3 Statistical analysis

Relative abundances of ASVs and phyla were calculated for fungi and bacteria for each sample type (i.e., rhizospheres and topsoils), after assessing the data for deviation from normality using the Shapiro–Wilk test. Diverging from the normal distribution, the Kruskal and Wallis (1952) and the *post hoc* Dunn’s test (Siegel, 1957; Siegel and Castellan, 1988) were implemented to analyse differences in relative abundances and alpha diversity values (phyla and observed ASVs richness, ASVs Shannon-Wiener index, and exponential Shannon-Wiener index) between each type of substrate (i.e., in this study, referred to the rhizospheres of different species and topsoil of the two plantations). The calculations were done using the “vegan” v2.6–8 (Oksanen et al., 2025), “psych” v2.5.6 (Revelle, 2025), and “microeco” v1.10.0 (Liu et al., 2021) packages with related dependencies in the R environment (R Core Team, 2024). The exponential Shannon-Weaver index (Hill number of order q = 1) was reported to display more intuitively the difference in diversity between substrates. This metric represents the number of equally abundant species (also referred to as “effective number of species”) required to achieve the same diversity as that assessed for each substrate (Chao et al., 2014).

Separate tests were conducted to evaluate community dissimilarities for fungal and bacterial communities, and these were also generally separated for samples belonging to the 2- and 10-year-old plantations. Still, comparisons were also conducted among the rhizospheres of all selected species, regardless of their provenance. The Jaccard distance based on incidence was adopted for its advantages over other distance metrics (see Chao et al., 2005). To assess the dissimilarities between fungal and bacterial community compositions, the Permutational Multivariate Analysis of Variance (PERMANOVA) was performed. Statistical difference between the relative abundance of the most abundant taxa among the rhizospheres and topsoil was calculated with the Kruskal–Wallis and Dunn’s *post hoc* tests, with *p*-value adjusted using the “fdr” method. We also ensured a careful discussion of the differences in microbial community composition by testing their variance. Given the unequal sample sizes (three samples for the rhizospheres and four for the topsoil), we compared the internal heterogeneity (i.e., variance or beta-diversity dispersion among samples) between groups with the Kruskal–Wallis and *post hoc* Dunn’s tests. To display dissimilarities between communities, Principal Coordinates Analysis (PCoA) was used, considering the calculated Jaccard distance, and Venn diagrams. To improve the readability of the results, the PCoA plots displayed three axes, using the package “plotly” v 4.12.0 (Sievert, 2020). All analyses were run using the “microeco” v1.10.0 (Liu et al., 2021) package.

Taxa characteristic of the different rhizospheres and topsoils were assessed through analysis of the indicator genus, using the “indicspecies” v1.8.0 (Cáceres and Legendre, 2009) package. The analysis was run separately for the communities of fungi and bacteria. The function (“multipatt”) was run considering each substrate separately (i.e., “duleg = T”), using the “r.g” parameter and default values. Resulting from this analysis is a set of genera associated with (i.e., indicators of) each substrate.

Statistical difference between relative abundances of the recognized functional groups among communities of the rhizospheres and of the topsoil was assessed with the Kruskal–Wallis and Dunn’s *post hoc* tests. All the statistical analyses were performed in the R environment (R Core Team, 2024). Code, metadata are visible at: https://github.com/JennyVivian99/Rhizospheres_Tropical_plantations.

## 3 Results

Following decontamination and quality checks, in the rhizospheres we found a total of 668,080 sequences and 3,604 observed ASVs (gamma diversity) for fungi, and 639,870 sequences with 24,820 observed ASVs for bacteria. Fungal analysis in the topsoil (of the 2- and 10-year-old plantation) resulted in a total of 506,728 sequences and 1828 observed ASVs, whereas 307,864 sequences and 11,985 observed ASVs were found for bacteria.

### 3.1 Fungal and bacterial richness of the rhizospheres and topsoil

The total number of ASVs was higher in the samples of the rhizosphere compared to those of the topsoil, but alpha diversity among rhizosphere and topsoil samples was not statistically different. Phylum richness of fungi and bacteria communities of each substrate did not differ significantly, as the standard and exponential Shannon-Wiener index of the observed ASVs (Kruskal–Wallis, *p* > 0.05) (Table 2).

**Table 2:** Alpha diversity of the analysed substrates.

	Soil/tree species	Fungi				Bacteria			
		Phyla richness	ASVs richness	Shannon–Wiener (ASVs)	Exponential Shannon index	Phyla richness	ASVs richness	Shannon–Wiener (ASVs)	Exponential Shannon index
2-year-old plantation	Topsoil	7.5 ± 1.3	266.2 ± 134	4.3 ± 0.2	74.3 ± 15.4	29 ± 2.8	1819.5 ± 103	6.8 ± 0.2	861.4 ± 124.8
	*A. mangium*	8.3 ± 1.5	302.3 ± 62.4	4 ± 0.7	64.7 ± 36.0	27.3 ± 0.6	1470.3 ± 310	6.3 ± 0.3	554.4 ± 147.7
	*Saccharum* spp.	8 ± 1	301.3 ± 40	4.1 ± 0.4	63.1 ± 20.3	31.7 ± 0.6	2036 ± 250	6.7 ± 0.2	801.3 ± 143.5
	*C. nucifera*	8 ± 1	269 ± 50	4.3 ± 0.2	75.8 ± 18.9	25.3 ± 6.1	1387.3 ± 547	6 ± 0.8	476.1 ± 290.5
	*I. cylindrica*	5.7 ± 0.6	155.3 ± 61	3.6 ± 0.3	36.8 ± 10.2	30.7 ± 1.5	2112.7 ± 272	6.7 ± 0.2	835.4 ± 129.8
10-year-old plantation	Topsoil	9.6 ± 0.5	308 ± 84.1	4.2 ± 0.3	71.2 ± 20.4	30.8 ± 1.3	1987 ± 330	6.6 ± 0.1	773.6 ± 110.7
	*A. mangium*	9 ± 0	303.3 ± 140	4.3 ± 0.3	77.1 ± 22.4	28.7 ± 2.1	1758.3 ± 166	6.4 ± 0.1	622.8 ± 70.4
	*P. indicus*	8.6 ± 0.6	166 ± 55	4 ± 0.3	54.8 ± 16.9	28.3 ± 1.5	1569.7 ± 37	6.5 ± 0.03	652.4 ± 18.8
	*S. macrophylla*	8 ± 1.4^‡^	311.5 ± 23^‡^	4 ± 0.02^‡^	53.9 ± 1^‡^	28.7 ± 2.5	1839 ± 100	6.6 ± 0.08	749.6 ± 62.8

Phyla and observed ASVs richness of fungi and bacteria communities of the different rhizospheres analysed, and the topsoil in which they occurred are reported along with standard and exponential Shannon-Wiener index on ASVs. For *S. macrophylla*, only two samples were analysed (indicated with “^‡^”), due to the low sequencing depth that led to the discarding of one tree. For the other plants, three samples were analysed, and 4 for topsoil. *A. mangium*, *Acacia mangium*; *C. nucifera*: *Cocos nucifera*; *I. cylindrica*: *Imperata cylindrica*; *P. indicus*: *Pterocarpus indicus*; *S. macrophylla*: *Swietenia macrophylla*.

### 3.2 Fungal beta-diversity among rhizospheres and topsoil

**Figure 2:**
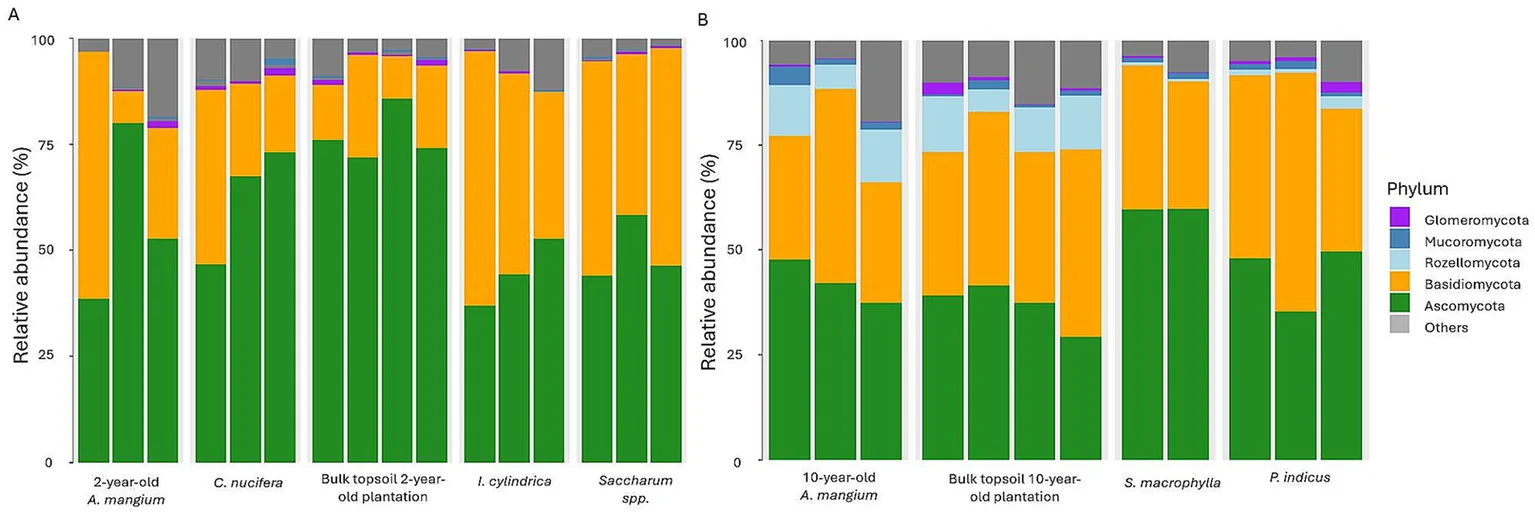
Relative abundances of fungal phyla for the 2-year-old **(A)** and 10-year-old **(B)** *Acacia mangium* plantation sites. *A. mangium*: *Acacia mangium*; *C. nucifera*: *Cocos nucifera*; *I. cylindrica*: *Imperata cylindrica*; *P. indicus*: *Pterocarpus indicus*; *S. macrophylla*: *Swietenia macrophylla*.

The composition of fungal communities did not differ among the analysed rhizospheres of eudicots and monocot plants, and respective topsoils (PERMANOVA, *p* > 0.05; Figures 2A,B; Supplementary Figure S5). In the 2-year-old plantation, fungi communities of the rhizospheres belonging to *I. cylindrica*, *C. nucifera*, *Saccharum* spp., and *A. mangium* showed a similar dispersion (i.e., Jaccard index evaluated through Kruskal–Wallis and Dunn’s test, *p* > 0.05). For the same metric, the topsoil held a comparable value to all the rhizospheres except with *I. cylindrica* (Kruskal–Wallis, *p* < 0.05). The variance of all fungal communities of the rhizospheres and topsoils of the 10-year-old plantation was not statistically different from each other (Kruskal–Wallis, *p* > 0.05 on Jaccard distances).

Ascomycota and Basidiomycota fungal phyla were dominant across all rhizospheres and topsoil samples (Figures 2A,B). In addition, Mortierellomycota, Kickxellomycota, and Chytridiomycota phyla were identified among the most dominant in the older plantation (although less abundant than those displayed in Figure 2). The Rozellomycota phylum displayed a slightly higher abundance in the rhizosphere of *A. mangium* and the topsoil of the 10-year-old stand (Figure 2B), but these did not differ significantly among the substrates (Kruskal–Wallis, *p* > 0.05).

**Figure 3:**
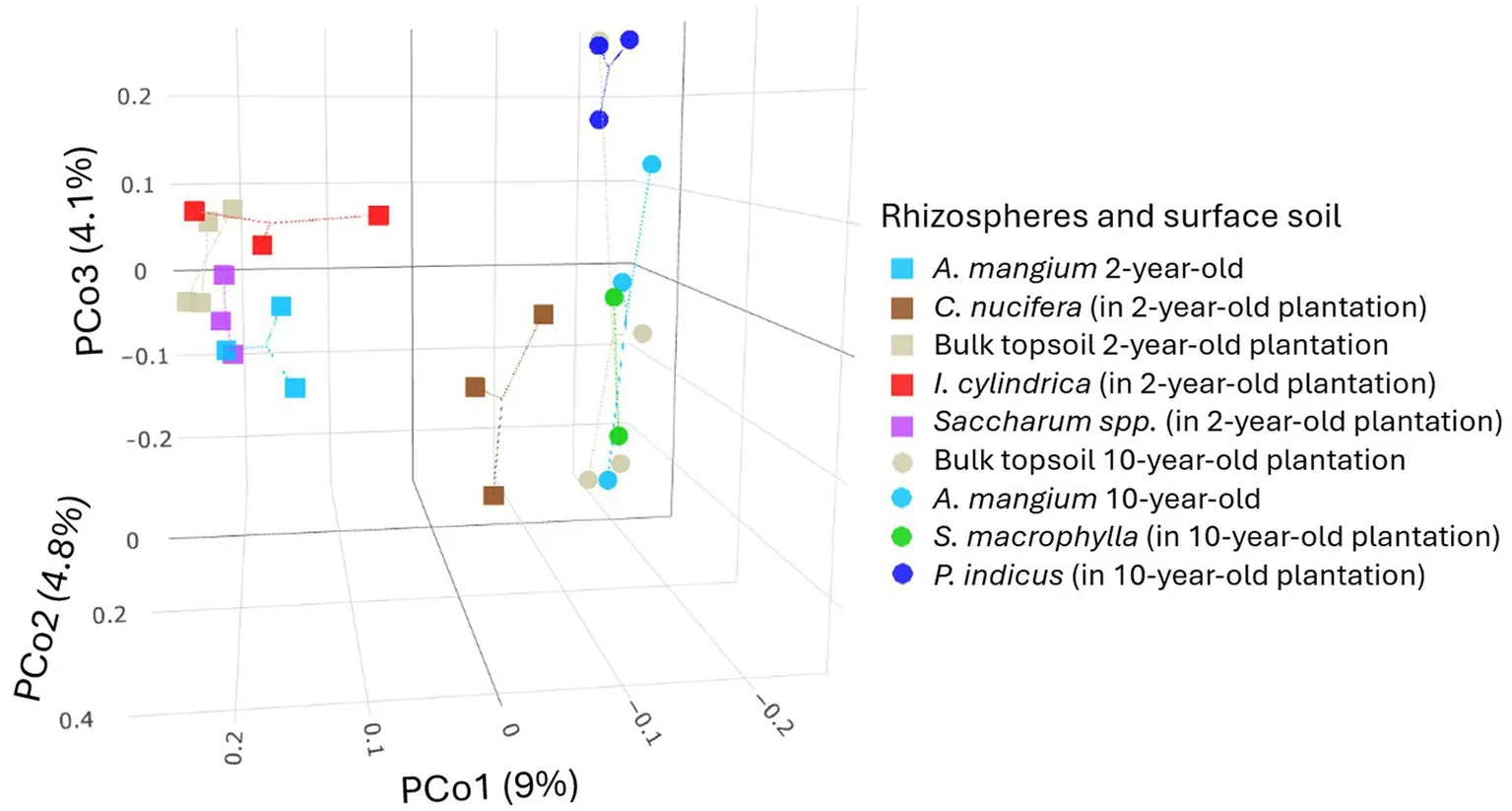
PCoA ordination of fungi of the rhizospheres and topsoil in the 2- and 10-year-old *Acacia mangium* plantations, based on Jaccard dissimilarity. PCoA: Principal coordinate analysis; PCo: Principal coordinate. *A. mangium*: *Acacia mangium*; *C. nucifera*: *Cocos nucifera*; *I. cylindrica*: *Imperata cylindrica*; *P. indicus*: *Pterocarpus indicus*; *S. macrophylla*: *Swietenia macrophylla*.

In the 2-year-old plantation, samples of the fungal communities of the topsoil did not differ from those of the rhizosphere of *I. cylindrica*, *Saccharum* spp., and *A. mangium* (PCoA; Figure 3). In contrast, the fungal community of *C. nucifera* rhizosphere clustered distinctively from those of the topsoil and the other species rhizospheres sampled. Samples from the 10-year-old plantation grouped without clear patterns (Figure 3). Of note is the distinct clustering between the fungal assemblages of the 2- and 10-year-old *A. mangium*, and among the fungal communities of the topsoil belonging to the two plantations.

Generally, one or two fungal genera characterized the substrates, allowing for a better understanding of their distinct traits (indicator species analysis; Table 3). Rhizospheres of *C. nucifera* and *S. macrophylla* were distinguishable from the others by three or more genera.

**Table 3:** Fungal genera characterizing the rhizospheres and topsoil of the analysed 2- and 10-year-old plantation, as calculated from the indicator species analysis.

Substrate	Genus
*A. mangium* 2-year-old	*Strelitziana*
*A. mangium* 10-year-old	*Bjerkandera*
Topsoil 2-year-old	*Capturomyces*
	*Acremoniopsis*
Topsoil 10-year-old	*Veronaeopsis*
*C. nucifera*	*Cladophialophora*
	*Silvaspora*
	*Tolypocladium*
*I. cylindrica*	*Komagataella*
	*Phaeoacremonium*
*S. macrophylla*	*Neodevriesia*
	*Amorocoelophoma*
	*Orbilia*
	*Terramyces*
	*Pseudochaetosphaeronema*
	*Hermetothecium*
	*Haudseptoria*
	*Metacordyceps*
	*Cryptocoryneum*
	*Clavulinopsis*
*P. indicus*	*Clitopilus*
	*Knufia*

*A. mangium*: *Acacia mangium*; *C. nucifera*: *Cocos nucifera*; *I. cylindrica*: *Imperata cylindrica*; *P. indicus*: *Pterocarpus indicus*; *S. macrophylla*: *Swietenia macrophylla*.

### 3.3 Bacterial beta-diversity among rhizospheres and topsoil

**Figure 4:**
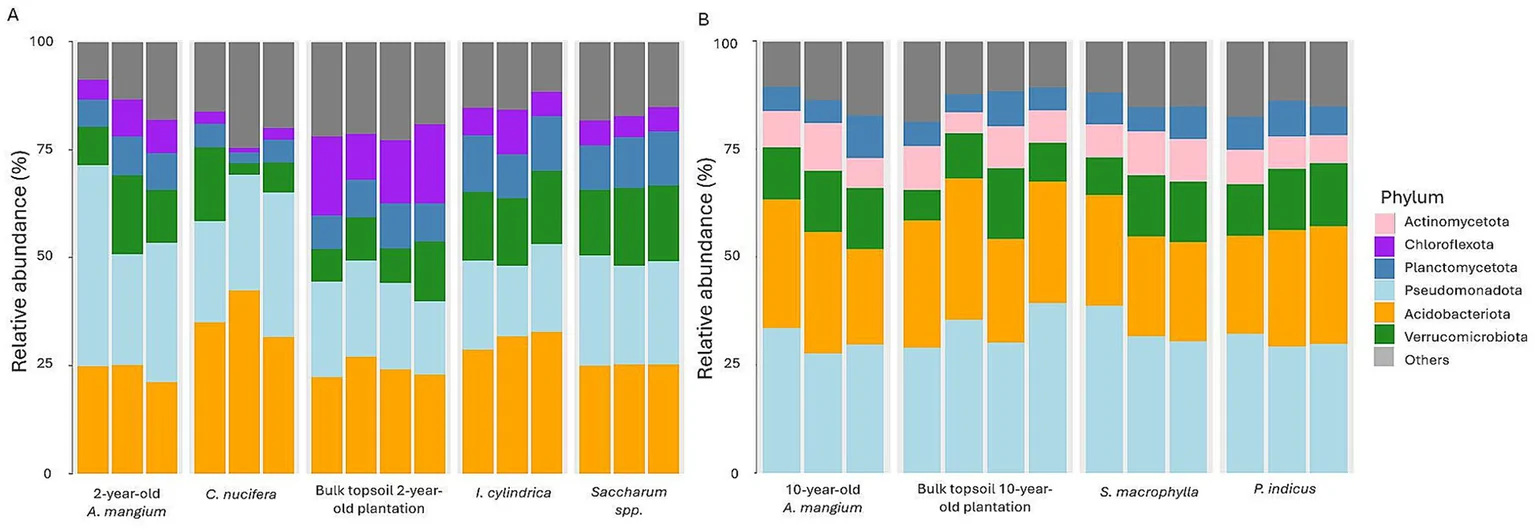
Relative abundances of bacterial phyla for the 2-year-old **(A)** and 10-year-old **(B)** *Acacia mangium* plantation sites. The phyla are ordinated from the most to the least abundant in each bar (i.e., sample). *A. mangium*: *Acacia mangium*; *C. nucifera*: *Cocos nucifera*; *I. cylindrica*: *Imperata cylindrica*; *P. indicus*: *Pterocarpus indicus*; *S. macrophylla*: *Swietenia macrophylla*.

In the 2- and 10-year-old plantation, rhizospheres of eudicots, monocots, and topsoil showed no statistically significant difference in bacterial community composition (PERMANOVA, *p* > 0.05; Figures 4A,B; Supplementary Figure S6). Bacteria of the rhizospheres and topsoil located in the 2-year-old plantation sites shared a similar dispersion. The exceptions were *Saccharum* spp. and *C. nucifera*, with the former expressing more variation than the latter (Kruskal–Wallis, *p* < 0.05 on Jaccard distances). In the 10-year-old plantation, bacterial communities of substrates displayed similar heterogeneity, except for *P. indicus* rhizosphere, which held a higher value of this metric compared to the topsoil (Kruskal–Wallis and Dunn’s test, *p* < 0.05).

Pseudomonadota, Acidobacteriota, Verrucomicrobiota, and Planctomycetota bacterial phyla were dominant in all the rhizospheres and topsoil samples analysed. Acidobacteriota was the most dominant phylum in the 2-year-old plantation, and Pseudomonadota in the 10-year-old stand (Figures 4A,B), but without a significant difference in their relative abundance among the analysed rhizospheres and topsoil. Chloroflexota was less abundant in the rhizosphere of *C. nucifera* compared to the topsoil of the 2-year-old plantation (Kruskal–Wallis, *p* < 0.05), but their value did not differ from that of the other rhizosphere samples from the same location (Kruskal–Wallis, *p* > 0.05). Moreover, the Chloroflexota phylum, widely present in the 2-year-old plantation, was uncommon in the communities of the 10-year-old one, and the opposite trend occurred for Actinomycetota.

**Figure 5:**
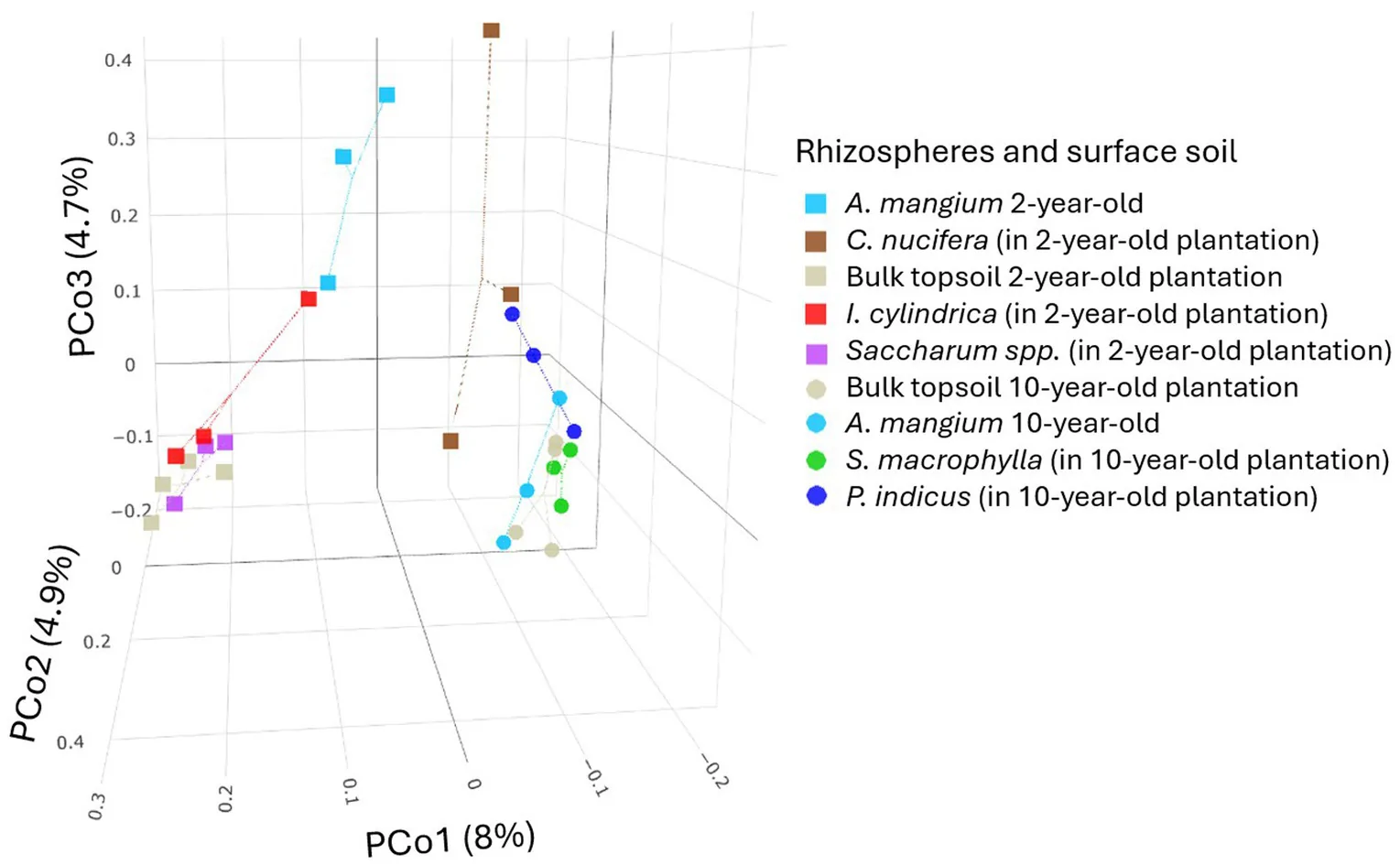
PCoA ordination of bacteria of the rhizospheres and topsoil in the 2- and 10-year-old plantations, based on Jaccard dissimilarity. PCoA: Principal coordinate analysis; PCo: Principal coordinate. *A. mangium: Acacia mangium*; *C. nucifera: Cocos nucifera*; *I. cylindrica: Imperata cylindrica*; *P. indicus: Pterocarpus indicus*; *S. macrophylla: Swietenia macrophylla*.

Samples of the bacterial communities from the topsoil of the 2-year-old plantation weakly clustered with those of the rhizospheres of *I. cylindrica* and *Saccharum* spp., but not with those of the 2-year-old *A. mangium* and *C. nucifera*. The samples of *C. nucifera* grouped separately from all the others (Figure 5). Samples of the 10-year-old plantation substrates generally clustered together, with the topsoil bacterial assemblages located close to those of the rhizospheres of the 10-year-old *A. mangium* and *P. indicus*. Similar to the fungal communities, samples of the 2- and 10-year-old *A. mangium* grouped separately from each other, and so did the topsoil of the two plantations.

Multiple bacterial genera characterised the analysed substrates, besides the rhizosphere of *I. cylindrica* and the 10-year-old *A. mangium*, for which no indicator genera were identified (here we report the names of a few taxa for each plant and topsoil; for the full list see Supplementary Table S1). The 2-year-old *A. mangium* rhizosphere was characterized by *Mesorhizobium* bacteria. *Gaiella*, and *Rhizomicrobium* distinguished the topsoil of the 2- and 10-year-old plantation, respectively. The bacterial community of the *C. nucifera* rhizosphere was indicated by the *Acidipila* genus. The *Rhizobium* genus characterized the rhizosphere of *Saccharum* spp., while *Leptothrix* and *Rhizorhabdus* genera distinguished the rhizosphere of *S. macrophylla*. Finally, worth noting is the genus *Candidatus Protochlamydia,* which characterized the *P. indicus* rhizosphere.

### 3.4 Fungal functional groups among rhizospheres and topsoil

Functional assignment resulted in the identification of fungal functions for 1,309 ASVs (47.7%) belonging to the communities of the topsoil and rhizospheres in the 2-year-old plantation, and for 1,025 ASVs (45.4%) for the same substrates in the 10-year-old plantation.

Functional traits of fungal communities generally differed among rhizospheres (Table 4). In the rhizosphere of *Saccharum* spp., the pathotrophic guild was particularly abundant, especially compared to the rhizosphere of *S. macrophylla*, from which it differed significantly. The saprotrophic group was largely present in the rhizospheres of *Saccharum* spp. and *I. cylindrica*, but not in that of the 10-year-old *A. mangium*. Ectomycorrhizal fungi were particularly abundant in the rhizosphere of *P. indicus* compared to the rhizospheres of *I. cylindrica* and *C. nucifera*.

**Table 4:** Relative abundance of fungi guilds and groups among the rhizospheres analysed.

		*Saccharum* spp.	10-year-old *A. mangium*	*S. macrophylla*	*P. indicus*	*C. nucifera*	*I. cylindrica*	2-year-old *A. mangium*
Guilds	Pathotroph	29.8 ± 10.6 a	15.9 ± 7.3 ab	12.9 ± 1.4 b	19.4 ± 12.4 ab	17.1 ± 6.9 ab	14.1 ± 5.4 ab	19.0 ± 5.9 ab
	Saprotroph	56.3 ± 6.9 a	35.3 ± 13.0 c	48.8 ± 3.9 abc	46.5 ± 9.0 abc	49.1 ± 16.2 abc	57.0 ± 8.1 ab	38.8 ± 8.7 bc
	Symbiotroph	30.0 ± 15.8 ab	26.0 ± 2.5 ab	20.3 ± 8.0 ab	29.7 ± 5.1 a	18.9 ± 9.7 ab	24.2 ± 13.6 ab	13.8 ± 9.2 b
Bryophyte Parasite		15.7 ± 11.0 a	1.0 ± 1.8 b	0.6 ± 0.9 b	5.3 ± 8.9 ab	3.7 ± 5.0 ab	4.5 ± 4.6 ab	4.8 ± 7.5 ab
Dung Saprotroph		16.3 ± 10.5 a	3.2 ± 2.0 ab	2.6 ± 2.8 b	7.0 ± 9.0 ab	8.0 ± 4.7 ab	7.5 ± 5.6 ab	6.1 ± 8.4 ab
Ectomycorrhizal		17.2 ± 10.7 abc	16.8 ± 2.5 ab	12.5 ± 8.9 abc	21.4 ± 6.0 a	6.6 ± 8.2 bc	5.1 ± 5.5 c	8.8 ± 6.8 abc
Fungal Parasite		26.8 ± 12.2 a	4.3 ± 2.1 b	3.2 ± 1.2 b	8.6 ± 9.6 ab	10.3 ± 7.0 ab	7.7 ± 5.7 ab	14.1 ± 9.4 ab
Leaf Saprotroph		16.3 ± 10.5 a	1.0 ± 1.8 b	0.7 ± 0.9 b	5.5 ± 8.8 ab	3.9 ± 4.8 ab	4.9 ± 5.5 ab	4.8 ± 7.5 ab
Plant Parasite		15.7 ± 11.0 a	1.0 ± 1.7 b	0.6 ± 0.9 b	5.5 ± 8.8 ab	3.7 ± 5.1 ab	4.4 ± 4.6 ab	4.8 ± 7.5 ab
Wood Saprotroph		20.8 ± 9.4 a	15.0 ± 10.1 a	11.8 ± 1.3 a	22.0 ± 11.3 a	18.4 ± 4.3 a	11.7 ± 8.4 a	8.6 ± 7.2 a
Animal Pathogen		10.3 ± 4.2 ab	5.6 ± 3.8 abc	4.0 ± 0.1 c	4.1 ± 0.2 bc	8.7 ± 2.7 abc	5.5 ± 2.3 abc	12.2 ± 4.0 a
Endophyte		3.0 ± 0.3 b	7.8 ± 3.9 ab	5.2 ± 0.4 ab	5.8 ± 0.7 ab	9.8 ± 5.3 a	4.5 ± 5.9 ab	3.4 ± 2.0 ab
Plant Pathogen		3.0 ± 1.8 a	7.8 ± 5.3 a	6.4 ± 1.3 a	6.8 ± 4.7 a	6.1 ± 3.9 a	3.9 ± 0.6 a	2.7 ± 0.9 a
Lichen Parasite		1.1 ± 0.5 a	1.4 ± 1.1 ab	0.4 ± 0.2 ab	0.4 ± 0.5 ab	1.2 ± 0.5 a	1.0 ± 1.1 ab	0.3 ± 0.1 b
Litter Saprotroph		0.3 ± 0.2 ab	0.3 ± 0.3 ab	0.4 ± 0.6 ab	0.3 ± 0.4 ab	0.7 ± 0.5 a	0.1 ± 0.1 b	0.4 ± 0.4 ab
Soil Saprotroph		1.5 ± 1.0 b	2.9 ± 2.9 ab	2.8 ± 1.5 ab	4.7 ± 1.8 a	5.8 ± 3.0 a	2.6 ± 1.4 ab	2.7 ± 1.8 ab
Plant Saprotroph		0.5 ± 0.6 b	2.8 ± 3.3 ab	2.2 ± 1.1 ab	2.4 ± 0.7 ab	5.3 ± 2.3 a	4.1 ± 6.8 ab	1.9 ± 1.8 ab
Epiphyte		0.2 ± 0.2 a	1.0 ± 1.1 a	0.7 ± 0.0 a	1.4 ± 1.1 a	0.2 ± 0.1 a	0.9 ± 1.6 a	0.1 ± 0.1 a
Lichenized		1.3 ± 2.2 a	0.1 ± 0.1 a	0.2 ± 0.2 a	0.2 ± 0.2 a	0.3 ± 0.6 a	0.1 ± 0.2 a	0.1 ± 0.1 a
Arbuscular Mycorrhizal		0.4 ± 0.2 ab	0.2 ± 0.2 b	0.4 ± 0.2 ab	1.4 ± 1.0 a	1.1 ± 0.6 a	0.3 ± 0.3 b	0.6 ± 0.8 ab
Endomycorrhizal		0.0 ± 0.0 a	0.0 ± 0.1 a	0.0 ± 0.0 a	0.0 ± 0.0 a	0.0 ± 0.0 a	0.0 ± 0.0 a	0.0 ± 0.0 a
Ericoid Mycorrhizal		0.0 ± 0.0 b	0.2 ± 0.2 ab	0.3 ± 0.4 ab	1.1 ± 1.0 a	0.0 ± 0.0 ab	0.0 ± 0.0 b	0.0 ± 0.0 b
Orchid Mycorrhizal		2.6 ± 1.4 a	0.1 ± 0.1 bcd	0.2 ± 0.1 abcd	0.0 ± 0.0 d	1.1 ± 1.0 abc	2.6 ± 3.7 ab	0.0 ± 0.0 cd
Clavicipitaceous Endophyte		0.0 ± 0.0 c	1.0 ± 1.3 a	0.0 ± 0.0 abc	0.0 ± 0.0 bc	0.5 ± 0.6 ab	0.0 ± 0.0 c	0.0 ± 0.0 c

Lowercase letters indicate the statistical difference among row values (Kruskal–Wallis and Dunn’s test, *p* < 0.05). *A. mangium*: *Acacia mangium*; *C. nucifera*: *Cocos nucifera*; *I. cylindrica*: *Imperata cylindrica*; *P. indicus*: *Pterocarpus indicus*; *S. macrophylla*: *Swietenia macrophylla*.

The relative abundance of fungi functional groups was mostly similar between 2- and 10-year-old *A. mangium* rhizospheres, but the Clavicipitaceous Endophyte showed a significantly higher relative abundance in the older trees (Table 4).

The topsoil of the 2-year-old plantation displayed a similar abundance of symbiotroph fungi compared to the rhizospheres of the plants present in the same site, except for *Saccharum* spp. and *I. cylindrica*, which showed a higher value of such guild (Supplementary Table S2). In the 10-year-old plantation, the topsoil and rhizospheres generally shared similar relative abundances of fungi functional groups (Supplementary Table S3). Still, we highlight the higher abundance of saprotrophs and arbuscular mycorrhizae in the *P. indicus* rhizosphere compared to the topsoil and 10-year-old *A. mangium* rhizosphere, respectively.

### 3.5 Bacterial functional groups among rhizospheres and topsoil

Possible functions were assigned for a total of 5,186 bacterial ASVs (26.83%), belonging to the communities present in the topsoil and rhizospheres of the 2-year-old plantation. For the samples from the 10-year-old plantation (topsoil and rhizospheres), the functions were identified for 4,635 ASVs (28.95%).

Relative abundances of bacterial functional groups were generally similar among rhizospheres (Supplementary Tables S4–S6). Relative abundances of bacterial functional groups were comparable between the rhizosphere of the 2- and 10-year-old *A. mangium* (Supplementary Table S4). In addition, the relative abundance of nitrogen fixers (average 0.1 ± 0) generally did not differ significantly among the rhizospheres.

## 4 Discussion

### 4.1 Complex dynamics may prompt similar topsoil microbial communities among rhizospheres

The unclear distinction between microbial communities belonging to eudicots and monocots disproved the first hypothesis on the taxonomic dissimilarity between the assemblages of the two groups based solely on their phylogenetic distance. Despite the higher richness of observed ASVs in the rhizospheres compared to the topsoil, the high variability of this value may have led to a potential similarity among the substrates. Indeed, even considering the effective number of microbial species, the diversity evaluated for each substrate was not statistically different, given a large spectrum of species abundance (e.g., 476.1 ± 290.5 for *C. nucifera*). Importantly, the small sample size and discarded sample potentially affected the detection of significant differences in microbial richness among rhizospheres and topsoil, stressing the importance of further analyses.

The microbial assemblages of *C. nucifera* that grouped distinctively from the others in the multidimensional space (Figures 3, 5) may suggest underlying differences in the filtering of the fungal and bacterial taxa between the rhizosphere of this species and the others (and the topsoil). The distinction of *C. nucifera* rhizosphere from the others by three fungal genera can potentially be an early indication of a different composition of *C. nucifera* rhizosphere compared to that of the topsoil, which may not have been detected by the analysis due to the experimental design (with samples of the rhizospheres not collected adjacent to those of the topsoil). Another factor possibly influencing the weak differentiation of the rhizosphere of *C. nucifera* from the other substrates is its differential abundance of Chloroflexota compared to the topsoil of the 2-year-old plantation. Besides potentially differentiating the microbial community of the rhizosphere of *C. nucifera* from the other substrates analysed, this result also partially sustains our expectation of a large relative abundance of such microbial taxa in monocots. The traits enabling *C. nucifera* to thrive in its native coastal ecosystems (Gunn et al., 2011; Beveridge et al., 2022) may have prompted such a difference in the microbial community composition of its rhizosphere. The results also potentially indicate a weaker role of the phylogenetic traits and ancestors compared to other drivers for the microbial filtering, such as ecological needs and niches of the plants.

The assessed lack of dissimilarity among assemblages of the rhizospheres can potentially indicate the role of topsoil as the main driver and pool for the microbial community composition (Bulgarelli et al., 2012). Subsequently, the analysed plant species may filter the taxa associated with their rhizosphere (Berg and Smalla, 2009). Supporting this hypothesis, previous studies reported that exudates produced by each plant species, root structure, and relationships with microorganisms for nutrient acquisition shape their preference for hosting certain microbial groups found in the soil reservoir (Badri et al., 2009; Dennis et al., 2010; Lakshmanan et al., 2012; Carvalhais et al., 2013; Lebeis et al., 2015; Zhou et al., 2025). Our results may be consistent with such a dynamic if a similar filtering of taxa is conducted among the analysed rhizospheres.

While the ecosystem modification (e.g., ecosystem structure, nutrient enrichments; Vivian et al., 2026a) reasonably influenced the microbial community composition of the topsoil (Vivian et al., 2026b), the taxonomic composition of the rhizospheres was generally unaffected. Besides the experimental design, and the small sample size, another reason for the non-significant difference in community composition found may be related to the widespread presence of root structures. As argued by Barberán et al. (2015), the microbial communities may still be distinct for each rhizosphere, but the extension of roots in the soil potentially hinders the recognition of their differences. In this context, the complex plant root system in the soil (Jenik, 1978; Tumber-Dávila et al., 2022) may cause their associated microbial communities to become indistinguishable from those of the other rhizospheres.

The Rozellomycota phylum, suggested to be fundamental in the carbon sink dynamics by decelerating decomposition (Gleason et al., 2012; Stüer-Patowsky et al., 2025) dominated the communities of the older plantation along with Ascomycota and Basidiomycota fungi. Being potentially associated with the Rozellomycota phylum, *A. mangium* may have supported its high abundance, possibly influencing the bio-sequestration of the system. While focused on soil bacteria, the recent study of Pereira et al. (2017) sustains the potentially large effect of *A. mangium* on the community composition of these microorganisms. Comparing the soil of monospecific and mixed stands of *A. mangium* and *Eucalyptus*, they (Gleason et al., 2012; Stüer-Patowsky et al., 2025) found a significant differentiation of the soil bacteria of the soil (0–100 cm) between the plantations containing *A. mangium* and those only occupied by *Eucalyptus*.

The taxonomic richness and community composition we found for the rhizosphere of each plant species, are generally consistent with previous studies. For example, the abundance of Pseudomonadota and Acidobacteriota we identified in the rhizosphere of *A. mangium* was also found by Yuan et al. (2022) (identifying OTUs). Our microbial diversity results (Shannon-Wiener index of ASVs) and abundance of Ascomycota and Pseudomonadota of the rhizosphere of *C. nucifera* reflect those of Bao et al. (2020). On the *Saccharum* spp. rhizosphere, we found a similar abundance of dominant fungi phyla to (Liu et al., 2023) (identifying fungi OTUs by sequencing 18S rRNA gene), even though they studied a commercial sugarcane variety. An additional study conducted on the rhizosphere of *Saccharum officinarum L.* displayed comparable abundances of microbial phyla to our study, except for Actinobacteria, for which the authors found a higher abundance with respect to our results (Li et al., 2022). The lack of extensive and comparative research undertaking high-throughput analysis of ITS and 16S rRNA genes for fungi and bacteria of the rhizosphere of *A. mangium* (particularly for the fungal community), *I. cylindrica*, *S. macrophylla*, and *P. indicus* in the field (but also in the laboratory) creates an obstacle to the generalization of our results. Acknowledging the influence of plants covering the area on the selection and growth of specific microbial taxa, studying the rhizosphere of these species is fundamental to understanding the restoration trajectory in plantations. Recognizing such a lack of information, this study helps improve the knowledge of the microbial assemblages of these plant species.

### 4.2 Fungal and bacterial functions in the rhizospheres and topsoil

Our hypotheses regarding the distinct rhizosphere functional profiles of eudicot and monocot plants were disproved, as the analysed rhizospheres shared similar putative functions profiles. Specifically, the predicted presence of symbiotrophic fungi displayed similar abundances among rhizospheres, and potential ectomycorrhizal fungi were not more abundant in the 10-year-old *A. mangium* compared to younger individuals of the same species. The predicted functional group of oligotrophic bacteria also dominated the communities of both monocots and dicots. In addition, and contrary to our expectation, fungi with potential arbuscular mycorrhiza functions had comparable high presence in the rhizospheres of eudicots and monocots (e.g., a large presence in *C. nucifera* and of *P. indicus*). However, the sample design, small sample size adopted, and the functional profile obtained from taxonomic information, rather than from the analyses of the expressed functions (through RNA sequencing), potentially affected the clarity of the results, limiting the detection of the differences between the functional profiles of microbes in the different rhizospheres and topsoils.

Interestingly, the rhizosphere of the older *A. mangium* was not characterised by predicted symbiotic bacteria. Again, a small sample size, and sampling *A. mangium* individuals of different dimensions in the 10-year-old plantation, possibly affected the study of the indicator genera (Table 1). In this case, taxa associated with the rhizosphere of mature trees could have been automatically discarded by the analysis because of their absence in the rhizosphere of the saplings evaluated in the same area, which did not reach full development compared to older individuals. Such a result becomes fundamental for forest restoration plans, exposing that the value of *A. mangium* as a N-fixing tree may vary throughout its growth.

Potentially indicating symbiotic relationships, the oligotrophic *Mesorhizobium* and *Rhizobium* genera characterised the 2-year-old *A. mangium* and *Saccharum* spp. rhizospheres, respectively. Importantly, the association of such microbial groups with the rhizosphere of *Saccharum* spp. and *A. mangium* only indicates the presence of such taxa in the proximity of the roots, but not the active exchange of nutrients. This is because the analyses were conducted by sampling the topsoil in the rhizosphere of the selected plants, without explicitly verifying the resource exchange between the organisms. Furthermore, sample sites were located on steep slopes. Hence, the presence of some microbial taxa may derive from downhill movement of the bacteria and not true symbiotic relationships, especially considering natural disturbances such as typhoons, resulting in heavy runoff. Still, in our case, the detection of *Mesorhizobium* and *Rhizobium* genera possibly indicates symbiotic relationships helping these plant species to thrive in harsh conditions (Dasgupta et al., 2021; El Idrissi et al., 2023), as the 2-year-old plantation is still morphologically a grassland dominated by *I. cylindrica*. The high abundance of ectomycorrhizal fungi found in the rhizosphere of *Saccharum* spp. in the 2-year-old plantation may be corroborating the presence of harsh conditions in this area.

The presence of fast-growing trees (i.e., *A. mangium*; Krisnawati et al., 2011) and established undergrowth (e.g., ferns, *A. mangium* saplings) makes the 10-year-old plantation a competitive environment for *P. indicus*, which was underplanted in the stand at four years. Indeed, being a relatively slow-growing tree in such conditions (Thomson, 2006), it exhibited a small stature (Table 1). Considering our results, it may be hypothesised that the abundance of the potential ectomycorrhizal fungal associations may enhance nutrient supply to *P. indicus* (Courty et al., 2010) in these plantations. While the beneficial role of arbuscular mycorrhizal (i.e., endosymbiotic) fungi for the growth of *P. indicus* has been extensively documented (e.g., Husna et al., 2021, 2022), the association of this tree species with the ectomycorrhiza group has been scarcely evaluated. Thus, our results provide a baseline for further research on the relationship between ectomycorrhizal fungi and *P. indicus* (but also other native species) in the context of forest regeneration and management.

### 4.3 Implications for forest restoration

Analyzing the microbial assemblages characterizing the rhizosphere of the dominant plant species can enhance the recognition of the potential effects of these species on ecosystem functions. By ensuring fungi and bacterial communities capable of promoting the growth of native species such as *P. indicus*, the restoration trajectory may be sustained along with a sufficient plantation yield for the local livelihood (e.g., through *A. mangium* occasional harvesting as in our study case; Vivian et al., 2026a).

Trees supporting a microbial community with a functional profile biased toward specific purposes (e.g., soil nutrients provision) should be further analysed for their potential role in forest restoration. In this context, the study of Bonner et al. (2019) highlights the potential of *S. macrophylla* for the restoration of soil microbial functions compared to logged native forests in the Philippines. Furthermore, focusing research efforts on the modification over time of the microbial community of *A. mangium* plantations may prevent the spread of native pests or diseases. Advances in the identification of the relationships between plants and soil microbes can potentially deepen the knowledge on the possible threats to biodiversity imposed by taxa of the Rozellomycota and Chytridiomycota phyla, known for including endoparasites for a variety of hosts. The role of occasional pioneer trees such as *C. nucifera* should also be considered, as the distinct traits of the microbial community of its rhizosphere may affect that of the surrounding soil and plants.

In our study, we implemented a space-for-time approach, considering a small number of replicates, which, in some cases, displayed a different variance of taxa abundances. Still, the results allowed us to explore the dynamics shaping the microbial communities of the rhizospheres, while recognizing specific taxa that can potentially benefit forest restoration and climate change mitigation plans.

## 5 Conclusion

Our preliminary study advances the knowledge of the microbial communities of the rhizosphere of *A. mangium, C. nucifera, Saccharum* spp.*, I. cylindrica, P. indicus,* and *S. macrophylla* in 2- and 10-year-old *A. mangium* plantations. Microbial assemblages of the rhizospheres of these dominant species and topsoils did not show statistically significant differences among species of eudicot and monocot plants, with the exception of *C. nucifera*. The profile of the putative functions expressed by fungi and bacteria also lacked statistically significant differences among the rhizospheres and topsoils. Recognizing the infancy of studies on the rhizosphere of cultivated and native plants in the tropics, our research serves as a baseline for future analyses focused on the identification of potential differences in the microbial composition of the rhizospheres of the analysed plants in different environmental conditions or with improved experimental settings. While being built on a limited sample design and sample size, it provides preliminary results on the possible similarity of microbial communities among the rhizospheres of distinct plant species, ultimately affecting ecosystem processes. By considering the potential influence of plant species on the local microbial community of the soil and related functions, the design of forest restoration plans can be improved, targeting the recovery of both above- and belowground aspects of the ecosystem.

## Data Availability

The datasets analysed for this study can be found in the NCBI Short Read Archive under the BioProject ID and accession number PRJNA1295595, while raw reads of the rhizosphere can be accessed through PRJNA1302026. Code and metadata are available at: https://github.com/JennyVivian99/Rhizospheres_Tropical_plantations.
