# Supplementary material for: Microbial rhizosphere and topsoil communities in young reforestation plantations
Source: Front Microbiol. 2026 Aug 26;17:1915742. doi: 10.3389/fmicb.2026.1915742 (PMC13553782; doi:10.3389/fmicb.2026.1915742)
Supplement: Supplementary file 1 [file Data_Sheet_1.PDF]

Supplementary Material

1 Figures

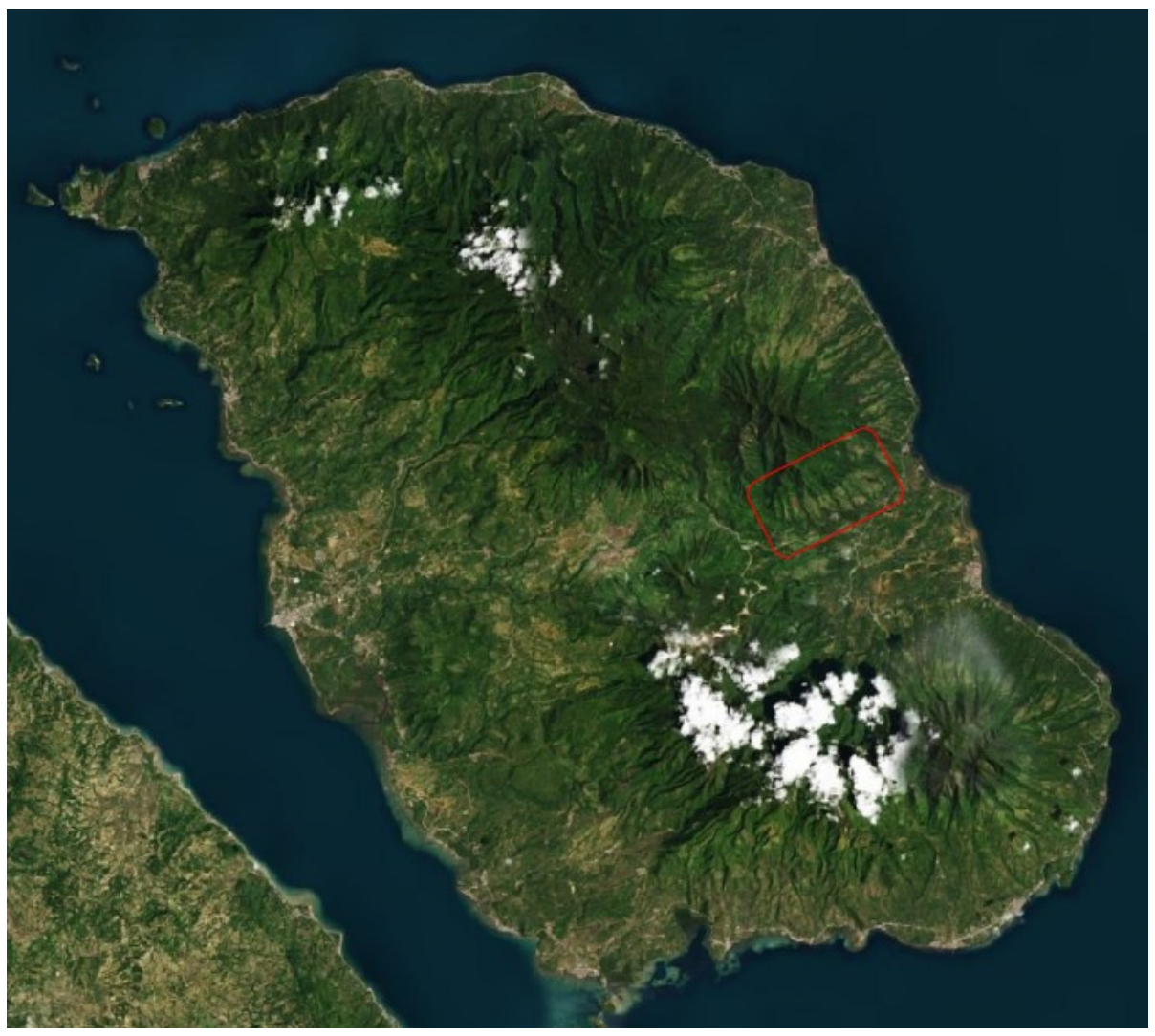

Fig. S1 Study site. Biliran, Philippines.

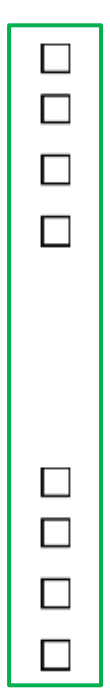

Fig. S2 Representation of the paired transects of the sampled plots in the 2- and 10-year-old plantation (bottom and upper transect, respectively), for the collection of the topsoil.

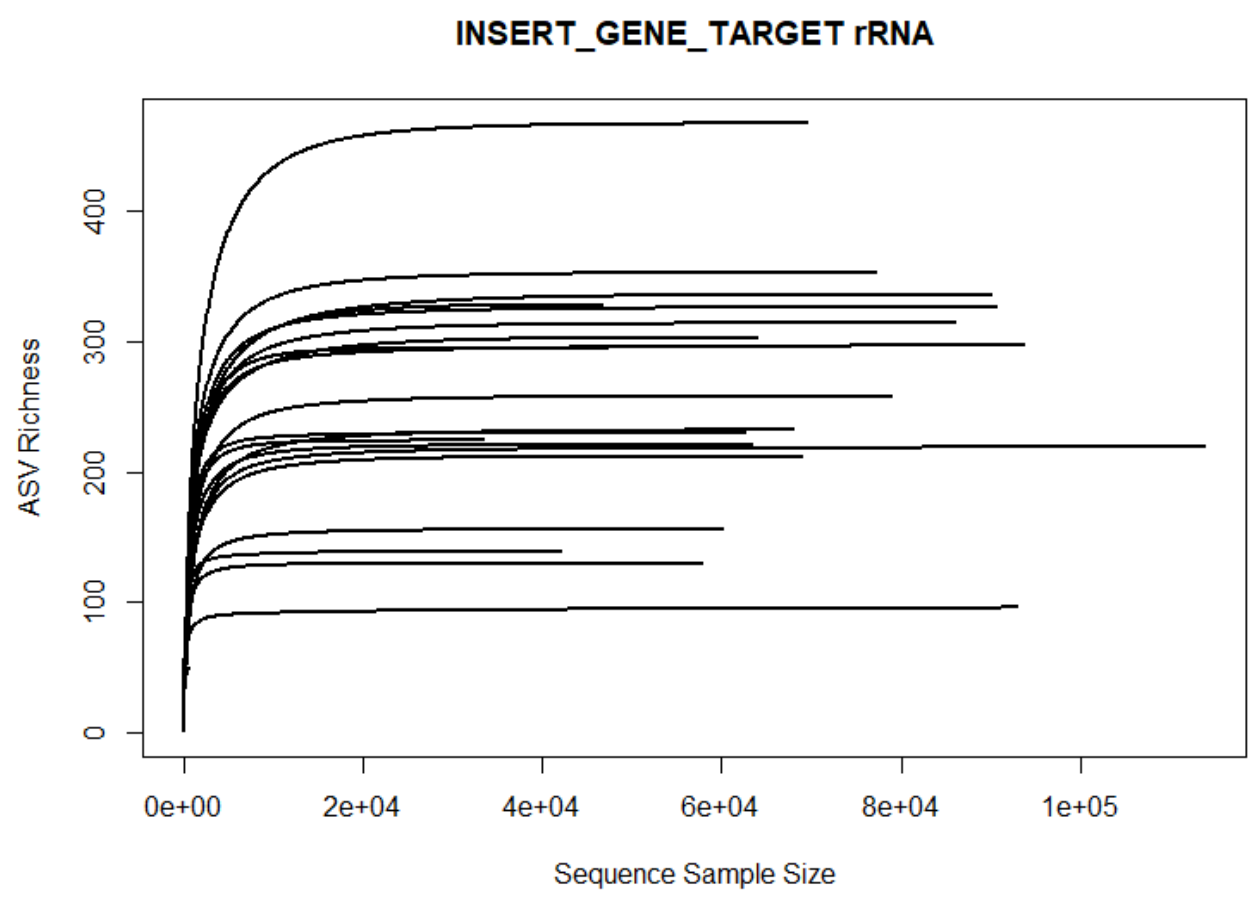

Fig. S3 Rarefaction curve of fungal sequences.

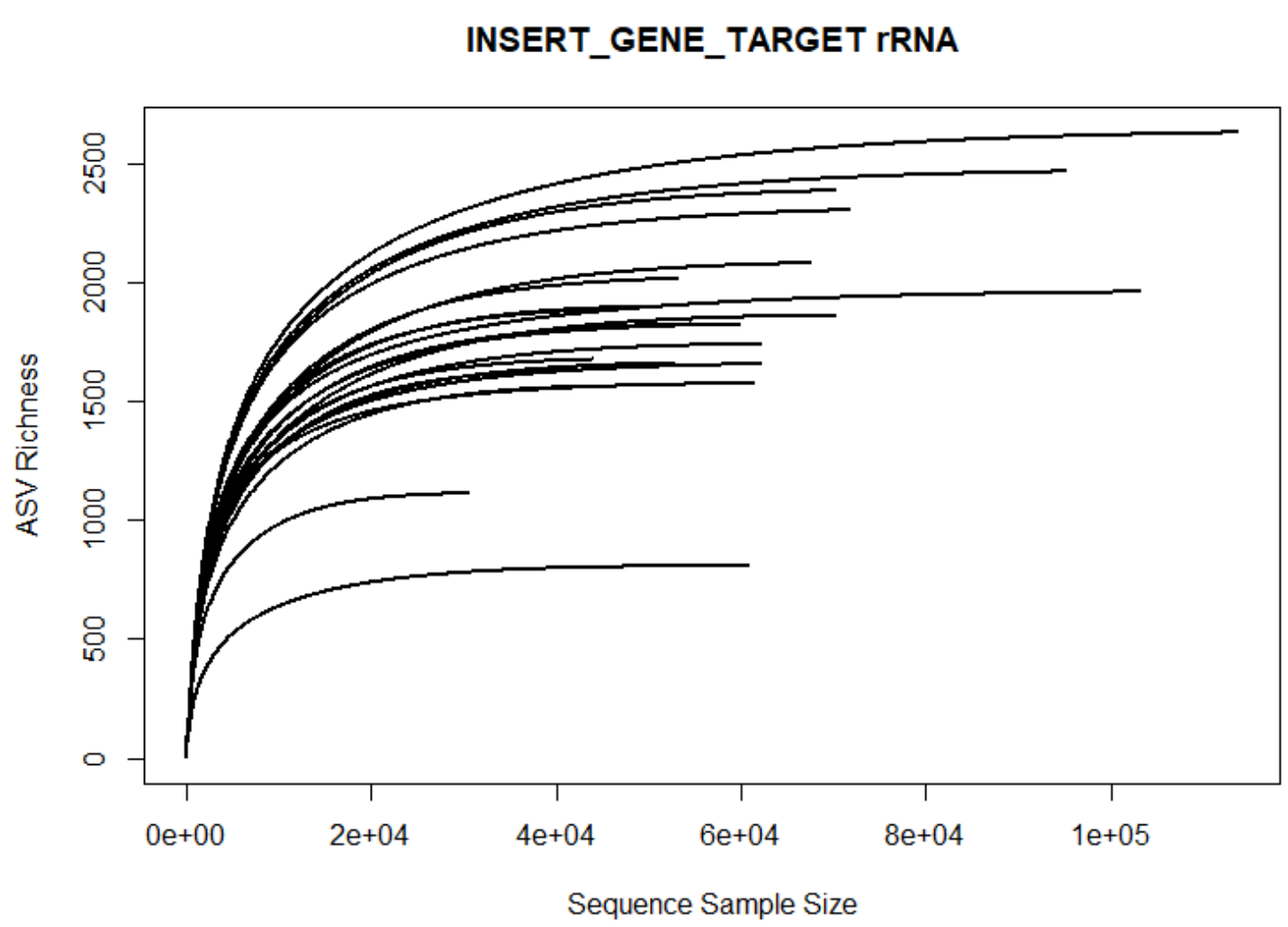

Fig. S4 Rarefaction curve of bacterial sequences.

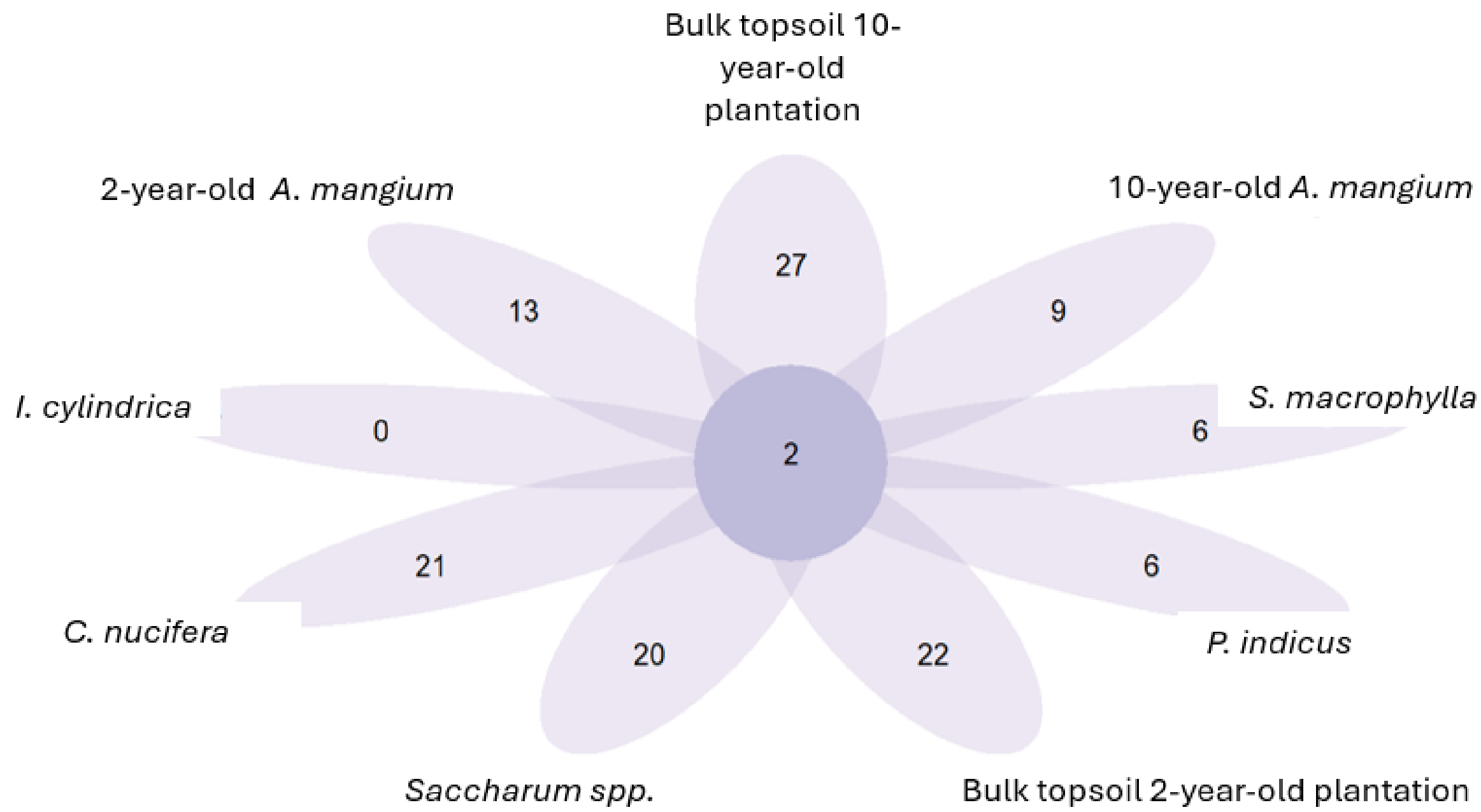

Fig. S5 Venn diagram of fungal ASVs. Filtered across groups, considering ASVs present at least in two samples per group.

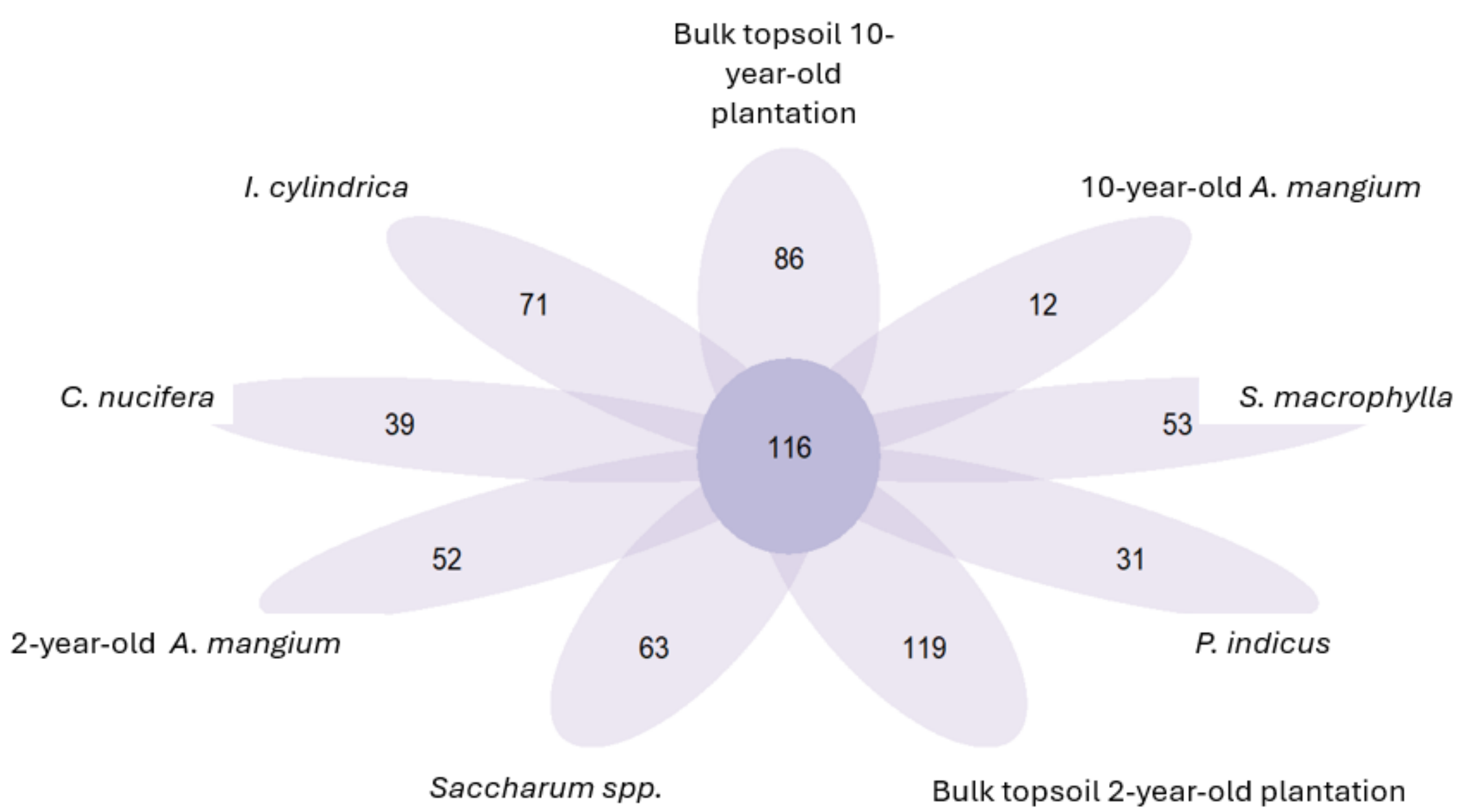

Fig. S6 Venn diagram of bacterial ASVs. Filtered across groups, considering ASVs present at least in two samples per group.

2 Tables

Table S1. Bacteria genera characterizing the rhizospheres and surface soil of the analysed 2- and 10-year-old plantation. No genera characterized the rhizosphere of the 10-year-old *A. mangium.*

Substrate	Genus
A. mangium 2-year-old	*Labrys*
	Ferrovum
	Mesorhizobium
	Candidatus Xiphinematobacter
	Hypericibacter
	Novosphingobium
	Nevskia
Surface soil 2-year-old	*Rhodoplanes*
	Rubrobacter
	MND1
	Amycolatopsis
	Allorhizocola
	Gaiella
	FCPS473
	Micromonospora
	Priestia
	Vicinamibacter
	Zavarzinella
	Crosiella
	1921-3
Surface soil 10-year-old	*Sporichthya*
	Dokdonella
	Oleiharenicola
	Rhizomicrobium
	Gemmatimonas
	Acidibacter
	Rhodovastum
C. nucifera	*Acidipila*
	Granulicella
	Luteibacter
	Candidatus Koribacter
	Acidicaldus
	Roseiarcus
	Occallatibacter
Saccharum spp.	*Rugosimonospora*
	Heliimonas
	Kineosporia
	Fimbriiglobus

	Ralstonia
	Azohydromonas
	Rhizobium
	Pirellula
	Dongia
S. macrophylla	*Paucibacter*
	Labrys
	LD29
	Tundrisphaera
	Aquabacterium
	Mucilaginibacter
	Pseudonocardia
	Leptothrix
	Rhizorhabdus
	alphaI cluster
	Spirosoma
	Novosphingobium
	Nevskia
	Duganella
	Lichenibacterium
	Cavicella
	Terriglobus
P. indicus	*Candidatus Protochlamydia*
	G12-WMSP1
	Vicinamibacter
	BacC-u-018

Table S2. Relative abundance of fungi functional groups in the rhizospheres and 2-year-old plantation topsoil.

	Topsoil 2-year-old plantation	*Saccharum spp.*	2-year-old *A. mangium*	*C. nucifera*	*I. cylindrica*
Pathotroph	20.19±3.75 ab	29.84±10.63 a	19.01±5.91 ab	17.09±6.92 ab	14.10±5.36 b
Saprotroph	39.91±9.61 a	56.34±6.94 a	38.81±8.73 a	49.12±16.16 a	57.01±8.09 a
Symbiotroph	6.84±1.56 b	30.01±15.78 a	13.76±9.20 ab	18.87±9.68 ab	24.25±13.56 a
Bryophyte Parasite	1.06±1.09 b	15.74±11.03 a	4.84±7.48 ab	3.74±5.01 ab	4.46±4.57 ab
Dung Saprotroph	2.65±1.38 b	16.34±10.45 a	6.12±8.42 ab	7.96±4.68 ab	7.52±5.59 ab
Ectomycorrhizal	2.21±1.31 b	17.21±10.73 a	8.79±6.80 ab	6.58±8.17 ab	5.08±5.54 ab
Fungal Parasite	17.81±4.33 ab	26.81±12.20 a	14.07±9.37 ab	10.28±7.00 ab	7.69±5.74 b
Leaf Saprotroph	1.07±1.08 b	16.27±10.47 a	4.84±7.48 ab	3.95±4.78 ab	4.93±5.50 ab
Plant Parasite	1.06±1.09 b	15.74±11.03 a	4.84±7.48 ab	3.69±5.05 ab	4.42±4.61 ab
Wood Saprotroph	8.96±3.33 a	20.84±9.37 a	8.62±7.22 a	18.40±4.28 a	11.65±8.36 a
Animal Pathogen	17.09±3.91 a	10.35±4.18 ab	12.18±4.03 ab	8.68±2.73 b	5.51±2.32 b
Endophyte	2.99±1.11 ab	2.98±0.32 ab	3.39±1.99 ab	9.78±5.34 a	4.54±5.89 b

Plant Pathogen	2.03±0.70 b	2.99±1.76 ab	2.72±0.91 ab	6.13±3.88 a	3.85±0.62 ab
Lichen Parasite	0.57±0.31 ab	1.12±0.49 a	0.30±0.06 b	1.21±0.50 a	1.04±1.08 ab
Litter Saprotroph	0.52±0.19 a	0.32±0.17 ab	0.43±0.35 ab	0.69±0.53 a	0.05±0.09 b
Soil Saprotroph	1.63±0.58 b	1.51±1.03 b	2.65±1.78 ab	5.84±3.03 a	2.59±1.41 ab
Plant Saprotroph	0.52±0.36 ab	0.54±0.60 b	1.89±1.82 ab	5.33±2.31 a	4.11±6.83 ab
Epiphyte	0.53±0.74 a	0.16±0.19 a	0.13±0.11 a	0.24±0.10 a	0.93±1.61 a
Lichenized	0.07±0.07 a	1.26±2.18 a	0.10±0.08 a	0.34±0.58 a	0.11±0.18 a
Arbuscular Mycorrhizal	0.89±0.48 ab	0.41±0.21 ab	0.63±0.77 ab	1.10±0.59 a	0.29±0.30 b
Endomycorrhizal	0.11±0.14 a	0.00±0.01 a	0.01±0.02 a	0.02±0.02 a	0.00±0.00 a
Ericoid Mycorrhizal	0.02±0.03 a	0.00±0.00 a	0.00±0.00 a	0.00±0.01 a	0.00±0.00 a
Orchid Mycorrhizal	0.56±1.01 ab	2.58±1.44 a	0.03±0.04 b	1.13±0.97 ab	2.63±3.67 ab
Clavicipitaceous Endophyte	0.04±0.04 ab	0.00±0.00 b	0.00±0.00 b	0.50±0.57 a	0.00±0.00 b

Table S3. Relative abundance of fungi functional groups in the rhizospheres and 10-year-old plantation topsoil.

	Topsoil 10-year-old plantation	10-year-old *A. mangium*	*S. macrophylla*	*P. indicus*
Pathotroph	14.9±2.2 a	15.9±7.3 a	12.9±1.4 a	19.4±12.4 a
Saprotroph	30.7±10.7 b	35.3±13.0 ab	48.8±3.9 ab	46.5±9.0 a
Symbiotroph	31.2±6.5 a	26.0±2.5 a	20.3±8.0 a	29.7±5.1 a
Bryophyte Parasite	0.3±0.1 a	1.0±1.8 a	0.6±0.9 a	5.3±8.9 a
Dung Saprotroph	1.9±1.1 a	3.2±2.0 a	2.6±2.8 a	7.0±9.0 a
Ectomycorrhizal	21.2±6.5 a	16.8±2.5 a	12.5±8.9 a	21.4±6.0 a
Fungal Parasite	4.5±1.5 a	4.3±2.1 a	3.2±1.2 a	8.6±9.6 a
Leaf Saprotroph	0.3±0.1 a	1.0±1.8 a	0.7±0.9 a	5.5±8.8 a
Plant Parasite	0.3±0.1 a	1.0±1.7 a	0.6±0.9 a	5.5±8.8 a
Wood Saprotroph	9.9±5.9 a	15.0±10.1 a	11.8±1.3 a	22.0±11.3 a
Animal Pathogen	3.8±0.3 a	5.6±3.8 a	4.0±0.1 a	4.1±0.2 a
Endophyte	7.6±1.2 a	7.8±3.9 a	5.2±0.4 a	5.8±0.7 a
Plant Pathogen	8.8±3.0 a	7.8±5.3 a	6.4±1.3 a	6.8±4.7 a
Lichen Parasite	0.4±0.3 a	1.4±1.1 a	0.4±0.2 a	0.4±0.5 a
Litter Saprotroph	1.2±0.8 a	0.3±0.3 a	0.4±0.6 a	0.3±0.4 a
Soil Saprotroph	2.4±2.6 a	2.9±2.9 a	2.8±1.5 a	4.7±1.8 a
Plant Saprotroph	1.2±0.7 a	2.8±3.3 a	2.2±1.1 a	2.4±0.7 a
Epiphyte	1.9±1.3 a	1.0±1.1 a	0.7±0.0 a	1.4±1.1 a
Lichenized	0.0±0.0 a	0.1±0.1 a	0.2±0.2 a	0.2±0.2 a
Arbuscular Mycorrhizal	1.1±1.2 ab	0.2±0.2 b	0.4±0.2 ab	1.4±1.0 a
Endomycorrhizal	0.3±0.3 a	0.0±0.1 a	0.0±0.0 a	0.0±0.0 a
Ericoid Mycorrhizal	0.0±0.1 a	0.2±0.2 a	0.3±0.4 a	1.1±1.0 a
Orchid Mycorrhizal	0.2±0.2 a	0.1±0.1 a	0.2±0.1 a	0.0±0.0 a
Clavicipitaceous Endophyte	0.2±0.3 ab	1.0±1.3 a	0.0±0.0 ab	0.0±0.0 b

Table S4. Relative abundance of bacterial functional groups in the different analysed rhizospheres.

	Saccharum *spp.*	2-year-old *A. mangium*	10-year-old *A. mangium*	*S. macrophylla*	*P. indicus*	*C. nucifera*	*I. cylindrica*
methanotrophy	0.0±0.0 a	0.0±0.0 a	0.0±0.0 a	0.0±0.0 a	0.0±0.0 a	0.1±0.0 a	0.0±0.0 a
methanol_oxidation	0.0±0.0 a	0.0±0.0 a	0.0±0.0 a	0.0±0.0 a	0.0±0.0 a	0.0±0.0 a	0.0±0.0 a
methylotrophy	0.0±0.0 a	0.0±0.0 a	0.0±0.0 a	0.0±0.0 a	0.0±0.0 a	0.1±0.0 a	0.0±0.0 a
aerobic_ammonia_oxidation	0.0±0.0 a	0.0±0.0 a	0.0±0.0 a	0.0±0.0 a	0.0±0.0 a	0.0±0.0 a	0.0±0.0 a
aerobic_nitrite_oxidation	0.0±0.0 a	0.0±0.0 a	0.0±0.0 a	0.0±0.0 a	0.0±0.0 a	0.0±0.1 a	0.0±0.0 a
nitrification	0.0±0.0 a	0.0±0.0 a	0.0±0.0 a	0.0±0.0 a	0.0±0.0 a	0.0±0.1 a	0.0±0.0 a
sulfate_respiration	0.3±0.1 a	0.4±0.1 a	0.5±0.2 a	0.6±0.3 a	0.6±0.2 a	0.0±0.0 a	0.4±0.2 a
sulfur_respiration	0.0±0.0 a	0.0±0.0 a	0.0±0.0 a	0.0±0.0 a	0.0±0.0 a	0.0±0.0 a	0.0±0.0 a
respiration_of_sulfur_compounds	0.3±0.1 a	0.4±0.1 a	0.5±0.2 a	0.6±0.3 a	0.6±0.2 a	0.0±0.0 a	0.4±0.2 a
anammox	0.0±0.0 a	0.0±0.0 a	0.0±0.0 a	0.0±0.0 a	0.0±0.0 a	0.0±0.0 a	0.0±0.0 a
nitrate_denitrification	0.0±0.0 a	0.0±0.0 a	0.0±0.0 a	0.0±0.0 a	0.0±0.0 a	0.0±0.0 a	0.0±0.0 a
nitrite_denitrification	0.0±0.0 a	0.0±0.0 a	0.0±0.0 a	0.0±0.0 a	0.0±0.0 a	0.0±0.0 a	0.0±0.0 a
denitrification	0.0±0.0 a	0.0±0.0 a	0.0±0.0 a	0.0±0.0 a	0.0±0.0 a	0.0±0.0 a	0.0±0.0 a
chitinolysis	0.0±0.0 a	0.0±0.0 a	0.0±0.0 a	0.0±0.0 a	0.0±0.0 a	0.1±0.2 a	0.0±0.0 a
dark_hydrogen_oxidation	6.2±0.7 a	13.7±8.6 a	12.3±3.6 a	10.5±2.7 a	11.5±1.8 a	7.3±2.3 a	5.2±0.6 a
nitrogen_fixation	0.1±0.0 a	0.1±0.1 a	0.0±0.0 a	0.1±0.1 a	0.0±0.0 a	0.1±0.0 a	0.0±0.0 a
nitrate_ammonification	0.0±0.0 a	0.0±0.0 a	0.0±0.0 a	0.0±0.0 a	0.0±0.0 a	0.0±0.0 a	0.0±0.0 a
nitrite_ammonification	0.0±0.0 a	0.0±0.0 a	0.0±0.0 a	0.0±0.0 a	0.0±0.0 a	0.0±0.0 a	0.0±0.0 a
nitrite_respiration	0.0±0.0 a	0.0±0.0 a	0.0±0.0 a	0.0±0.0 a	0.0±0.0 a	0.0±0.0 a	0.0±0.0 a
cellulolysis	1.3±0.1 a	1.9±0.6 a	1.6±0.7 a	2.3±1.2 a	2.1±0.1 a	3.3±2.5 a	1.2±0.4 a
xylanolysis	0.1±0.0 a	0.1±0.0 a	0.1±0.0 a	0.0±0.0 a	0.1±0.0 a	0.3±0.2 a	0.0±0.0 a
dark_thiosulfate_oxidation	0.0±0.0 a	0.0±0.0 a	0.0±0.0 a	0.0±0.0 a	0.0±0.0 a	0.0±0.1 a	0.0±0.0 a
dark_oxidation_of_sulfur_compounds	0.0±0.0 a	0.0±0.0 a	0.0±0.0 a	0.0±0.0 a	0.0±0.0 a	0.0±0.1 a	0.0±0.0 a
manganese_oxidation	0.0±0.0 ab	0.0±0.0 ab	0.0±0.0 b	0.1±0.1 a	0.0±0.0 ab	0.0±0.0 ab	0.0±0.0 ab
manganese_respiration	0.0±0.0 a	0.0±0.0 a	0.0±0.0 a	0.0±0.0 a	0.0±0.0 a	0.0±0.0 a	0.0±0.0 a
fermentation	0.3±0.2 a	0.1±0.1 a	0.3±0.4 a	0.3±0.3 a	0.2±0.1 a	0.7±0.4 a	0.2±0.1 a
aerobic_chemoheterotrophy	21.8±1.6 a	31.7±9.5 a	27.1±5.6 a	28.7±2.2 a	26.7±2.0 a	28.9±7.6 a	19.1±1.9 a
invertebrate_parasites	0.0±0.0 a	0.3±0.4 a	0.1±0.0 a	0.1±0.0 a	0.2±0.1 a	0.1±0.1 a	0.0±0.0 a
human_pathogens_pneumonia	0.1±0.1 a	0.0±0.1 a	0.0±0.0 a	0.2±0.2 a	0.0±0.0 a	0.2±0.3 a	0.0±0.0 a
human_pathogens_nosocomia	0.0±0.1 a	0.0±0.0 a	0.0±0.0 a	0.0±0.0 a	0.0±0.0 a	0.0±0.0 a	0.0±0.0 a
human_pathogens_all	0.2±0.1 a	0.4±0.5 a	0.3±0.3 a	0.5±0.2 a	0.3±0.1 a	0.8±0.9 a	0.2±0.1 a
human_gut	0.0±0.0 a	0.0±0.0 a	0.0±0.0 a	0.0±0.0 a	0.0±0.0 a	0.1±0.2 a	0.0±0.0 a
human_associated	0.3±0.2 a	0.4±0.5 a	0.3±0.3 a	0.5±0.2 a	0.3±0.1 a	0.9±1.1 a	0.2±0.1 a
mammal_gut	0.0±0.0 a	0.0±0.0 a	0.0±0.0 a	0.0±0.0 a	0.0±0.0 a	0.1±0.2 a	0.0±0.0 a
animal_parasites_or_symbionts	2.5±0.1 a	4.7±0.8 a	2.6±1.4 a	5.6±2.3 a	4.6±0.8 a	2.5±2.0 a	2.4±1.4 a
oil_bioremediation	0.0±0.0 a	0.0±0.0 a	0.0±0.0 a	0.0±0.0 a	0.0±0.0 a	0.0±0.0 a	0.0±0.0 a
aromatic_hydrocarbon_degradation	0.1±0.2 a	0.1±0.1 a	0.0±0.0 a	0.1±0.1 a	0.1±0.0 a	0.1±0.1 a	0.0±0.0 a
aromatic_compound_degradation	0.3±0.2 a	0.1±0.1 a	0.1±0.0 a	0.2±0.2 a	0.1±0.0 a	0.4±0.5 a	0.0±0.0 a

aliphatic_non_methane_hydrocarbon_degradation	0.0±0.0 a	0.0±0.0 a	0.0±0.0 a	0.0±0.0 a	0.0±0.0 a	0.1±0.1 a	0.0±0.0 a
hydrocarbon_degradation	0.2±0.2 a	0.1±0.1 a	0.0±0.0 a	0.1±0.1 a	0.1±0.0 a	0.1±0.2 a	0.1±0.0 a
iron_respiration	0.2±0.0 a	0.2±0.2 a	0.4±0.1 a	0.4±0.3 a	0.4±0.1 a	1.5±1.6 a	0.2±0.1 a
nitrate_respiration	0.0±0.0 a	0.0±0.0 a	0.1±0.1 a	0.2±0.1 a	0.1±0.0 a	0.3±0.4 a	0.0±0.0 a
nitrate_reduction	0.4±0.2 a	0.8±1.1 a	0.9±0.8 a	0.9±0.4 a	0.7±0.2 a	0.9±1.1 a	0.3±0.1 a
nitrogen_respiration	0.0±0.0 a	0.0±0.0 a	0.1±0.1 a	0.2±0.1 a	0.1±0.0 a	0.3±0.4 a	0.0±0.0 a
fumarate_respiration	0.0±0.0 a	0.0±0.0 a	0.0±0.0 a	0.0±0.0 a	0.0±0.0 a	0.0±0.0 a	0.0±0.0 a
intracellular_parasites	0.3±0.1 a	0.3±0.1 a	0.4±0.2 a	0.5±0.2 a	0.7±0.4 a	0.4±0.1 a	0.7±0.4 a
chlorate_reducers	0.0±0.0 a	0.0±0.0 a	0.0±0.0 a	0.0±0.0 a	0.0±0.0 a	0.0±0.0 a	0.0±0.0 a
predatory_or_exoparasitic	0.1±0.1 a	0.1±0.2 a	0.1±0.2 a	0.1±0.0 a	0.0±0.0 a	0.0±0.0 a	0.1±0.1 a
chloroplasts	0.1±0.0 a	0.4±0.4 a	0.5±0.4 a	0.7±0.6 a	0.7±0.9 a	1.8±0.7 a	0.2±0.2 a
photosynthetic_cyanobacteria	0.1±0.0 a	0.4±0.4 a	0.5±0.4 a	0.7±0.6 a	0.7±0.9 a	1.8±0.7 a	0.2±0.2 a
anoxygenic_photoautotrophy_S_oxidizing	0.0±0.0 a	0.0±0.0 a	0.0±0.0 a	0.0±0.0 a	0.0±0.0 a	0.0±0.0 a	0.0±0.0 a
anoxygenic_photoautotrophy	0.0±0.0 a	0.0±0.0 a	0.0±0.0 a	0.0±0.0 a	0.0±0.0 a	0.0±0.0 a	0.0±0.0 a
oxygenic_photoautotrophy	0.1±0.0 a	0.4±0.4 a	0.5±0.4 a	0.7±0.6 a	0.7±0.9 a	1.8±0.7 a	0.2±0.2 a
photoautotrophy	0.1±0.0 a	0.4±0.5 a	0.5±0.4 a	0.7±0.6 a	0.7±0.9 a	1.8±0.8 a	0.2±0.2 a
aerobic_anoxygenic_phototrophy	0.0±0.0 a	0.0±0.0 a	0.0±0.0 a	0.0±0.0 a	0.0±0.0 a	0.0±0.0 a	0.0±0.0 a
photoheterotrophy	0.0±0.0 a	0.0±0.0 a	0.0±0.0 a	0.0±0.0 a	0.0±0.0 a	0.1±0.1 a	0.0±0.0 a
phototrophy	0.1±0.0 a	0.4±0.5 a	0.5±0.4 a	0.7±0.5 a	0.7±0.9 a	1.9±0.8 a	0.2±0.2 a
plastic_degradation	0.1±0.1 a	0.0±0.1 a	0.0±0.0 a	0.0±0.1 a	0.0±0.0 a	0.0±0.0 a	0.0±0.0 a
ureolysis	0.3±0.1 a	0.1±0.2 a	0.2±0.2 a	0.2±0.0 a	0.3±0.1 a	0.3±0.3 a	0.2±0.0 a
chemoheterotrophy	26.0±2.3 a	33.6±8.8 a	30.9±3.7 a	31.7±2.1 a	29.5±1.3 a	31.0±7.3 a	23.6±1.6 a
anaerobic_chemoheterotrophy	4.2±0.7 a	1.9±0.8 a	3.8±2.3 a	3.0±1.2 a	2.8±0.8 a	2.2±1.1 a	4.5±0.9 a

Table S5. Relative abundances of bacterial functional groups in the rhizospheres and 2-year-old plantation topsoil.

	2-year-old *A. mangium*	*C. nucifera*	Topsoil 2-year-old plantation	*I. cylindrica*	*Saccharum spp.*
methanotrophy	0.0±0.0 a	0.1±0.0 a	0.0±0.0 a	0.0±0.0 a	0.0±0.0 a
methylotrophy	0.0±0.0 a	0.1±0.0 a	0.0±0.0 a	0.0±0.0 a	0.0±0.0 a
aerobic_ammonia_oxidation	0.0±0.0 a	0.0±0.0 a	0.0±0.0 a	0.0±0.0 a	0.0±0.0 a
aerobic_nitrite_oxidation	0.0±0.0 a	0.0±0.1 a	0.0±0.0 a	0.0±0.0 a	0.0±0.0 a
nitrification	0.0±0.0 a	0.0±0.1 a	0.0±0.0 a	0.0±0.0 a	0.0±0.0 a
sulfate_respiration	0.4±0.1 a	0.0±0.0 b	0.4±0.4 ab	0.4±0.2 a	0.3±0.1 ab
respiration_of_sulfur_compounds	0.4±0.1 a	0.0±0.0 b	0.4±0.4 ab	0.4±0.2 a	0.3±0.1 ab
anammox	0.0±0.0 a	0.0±0.0 a	0.0±0.0 a	0.0±0.0 a	0.0±0.0 a
nitrate_denitrification	0.0±0.0 a	0.0±0.0 a	0.0±0.0 a	0.0±0.0 a	0.0±0.0 a
nitrite_denitrification	0.0±0.0 a	0.0±0.0 a	0.0±0.0 a	0.0±0.0 a	0.0±0.0 a
denitrification	0.0±0.0 a	0.0±0.0 a	0.0±0.0 a	0.0±0.0 a	0.0±0.0 a
chitinolysis	0.0±0.0 a	0.1±0.2 a	0.0±0.0 a	0.0±0.0 a	0.0±0.0 a
dark_hydrogen_oxidation	13.7±8.6 a	7.3±2.3 ab	7.0±0.5 a	5.2±0.6 b	6.2±0.7 ab

nitrogen_fixation	0.1±0.1 ab	0.1±0.0 ab	0.0±0.0 b	0.0±0.0 ab	0.1±0.0 a
nitrate_ammonification	0.0±0.0 a	0.0±0.0 a	0.0±0.0 a	0.0±0.0 a	0.0±0.0 a
nitrite_ammonification	0.0±0.0 a	0.0±0.0 a	0.0±0.0 a	0.0±0.0 a	0.0±0.0 a
nitrite_respiration	0.0±0.0 ab	0.0±0.0 a	0.0±0.0 b	0.0±0.0 ab	0.0±0.0 ab
cellulolysis	1.9±0.6 a	3.3±2.5 a	1.4±0.5 a	1.2±0.4 a	1.3±0.1 a
xylanolysis	0.1±0.0 ab	0.3±0.2 a	0.0±0.0 bc	0.0±0.0 c	0.1±0.0 bc
dark_thiosulfate_oxidation	0.0±0.0 ab	0.0±0.1 a	0.0±0.0 b	0.0±0.0 b	0.0±0.0 b
dark_oxidation_of_sulfur_compounds	0.0±0.0 ab	0.0±0.1 a	0.0±0.0 b	0.0±0.0 b	0.0±0.0 b
manganese_oxidation	0.0±0.0 ab	0.0±0.0 ab	0.0±0.0 b	0.0±0.0 ab	0.0±0.0 a
fermentation	0.1±0.1 bc	0.7±0.4 a	0.1±0.1 c	0.2±0.1 abc	0.3±0.2 ab
aerobic_chemoheterotrophy	31.7±9.5 a	28.9±7.6 a	19.7±0.1 b	19.1±1.9 b	21.8±1.6 ab
invertebrate_parasites	0.3±0.4 ab	0.1±0.1 a	0.1±0.1 ab	0.0±0.0 b	0.0±0.0 ab
human_pathogens_pneumonia	0.0±0.1 a	0.2±0.3 a	0.0±0.0 a	0.0±0.0 a	0.1±0.1 a
human_pathogens_nosocomia	0.0±0.0 a	0.0±0.0 a	0.0±0.0 a	0.0±0.0 a	0.0±0.1 a
human_pathogens_all	0.4±0.5 a	0.8±0.9 a	0.1±0.1 a	0.2±0.1 a	0.2±0.1 a
human_gut	0.0±0.0 a	0.1±0.2 a	0.0±0.0 a	0.0±0.0 a	0.0±0.0 a
human_associated	0.4±0.5 a	0.9±1.1 a	0.1±0.1 a	0.2±0.1 a	0.3±0.2 a
mammal_gut	0.0±0.0 a	0.1±0.2 a	0.0±0.0 a	0.0±0.0 a	0.0±0.0 a
animal_parasites_or_symbionts	4.7±0.8 a	2.5±2.0 ab	0.8±0.3 b	2.4±1.4 ab	2.5±0.1 ab
oil_bioremediation	0.0±0.0 a	0.0±0.0 a	0.0±0.0 a	0.0±0.0 a	0.0±0.0 a
aromatic_hydrocarbon_degradation	0.1±0.1 a	0.1±0.1 a	0.0±0.0 a	0.0±0.0 a	0.1±0.2 a
aromatic_compound_degradation	0.1±0.1 ab	0.4±0.5 ab	0.1±0.0 ab	0.0±0.0 b	0.3±0.2 a
aliphatic_non_methane_hydrocarbon_degradation	0.0±0.0 a	0.1±0.1 a	0.0±0.0 a	0.0±0.0 a	0.0±0.0 a
hydrocarbon_degradation	0.1±0.1 a	0.1±0.2 a	0.0±0.0 a	0.1±0.0 a	0.2±0.2 a
iron_respiration	0.2±0.2 ab	1.5±1.6 a	0.1±0.1 b	0.2±0.1 b	0.2±0.0 b
nitrate_respiration	0.0±0.0 a	0.3±0.4 a	0.0±0.0 a	0.0±0.0 a	0.0±0.0 a
nitrate_reduction	0.8±1.1 a	0.9±1.1 a	0.3±0.2 a	0.3±0.1 a	0.4±0.2 a
nitrogen_respiration	0.0±0.0 a	0.3±0.4 a	0.0±0.0 a	0.0±0.0 a	0.0±0.0 a
fumarate_respiration	0.0±0.0 a	0.0±0.0 a	0.0±0.0 a	0.0±0.0 a	0.0±0.0 a
intracellular_parasites	0.3±0.1 a	0.4±0.1 a	0.3±0.2 a	0.7±0.4 a	0.3±0.1 a
chlorate_reducers	0.0±0.0 a	0.0±0.0 a	0.0±0.0 a	0.0±0.0 a	0.0±0.0 a
predatory_or_exoparasitic	0.1±0.2 a	0.0±0.0 a	0.0±0.0 a	0.1±0.1 a	0.1±0.1 a
chloroplasts	0.4±0.4 ab	1.8±0.7 a	0.3±0.4 ab	0.2±0.2 b	0.1±0.0 b
photosynthetic_cyanobacteria	0.4±0.4 ab	1.8±0.7 a	0.3±0.3 ab	0.2±0.2 b	0.1±0.0 b
anoxygenic_photoautotrophy_S_oxidizing	0.0±0.0 a	0.0±0.0 a	0.0±0.0 a	0.0±0.0 a	0.0±0.0 a
anoxygenic_photoautotrophy	0.0±0.0 a	0.0±0.0 a	0.0±0.0 a	0.0±0.0 a	0.0±0.0 a
oxygenic_photoautotrophy	0.4±0.4 ab	1.8±0.7 a	0.3±0.3 ab	0.2±0.2 b	0.1±0.0 b
photoautotrophy	0.4±0.5 ab	1.8±0.8 a	0.3±0.3 ab	0.2±0.2 b	0.1±0.0 b
aerobic_anoxygenic_phototrophy	0.0±0.0 a	0.0±0.0 a	0.0±0.0 a	0.0±0.0 a	0.0±0.0 a
photoheterotrophy	0.0±0.0 ab	0.1±0.1 a	0.0±0.0 b	0.0±0.0 ab	0.0±0.0 ab
phototrophy	0.4±0.5 ab	1.9±0.8 a	0.4±0.4 b	0.2±0.2 b	0.1±0.0 b
plastic_degradation	0.0±0.1 a	0.0±0.0 a	0.0±0.0 a	0.0±0.0 a	0.1±0.1 a
ureolysis	0.1±0.2 a	0.3±0.3 a	0.1±0.1 a	0.2±0.0 a	0.3±0.1 a
chemoheterotrophy	33.6±8.8 a	31.0±7.3 a	24.9±1.8 ab	23.6±1.6 b	26.0±2.3 ab

anaerobic_chemoheterotrophy	1.9±0.8 b	2.2±1.1 b	5.1±1.7 a	4.5±0.9 a	4.2±0.7 ab

Table S6. Relative abundances of bacterial functional groups in the rhizospheres and 10-year-old plantation topsoil.

	10-year-old *A. mangium*	Topsoil 10-year-old plantation	*S. macrophylla*	*P. indicus*
methanotrophy	0.0±0.0 a	0.0±0.0 a	0.0±0.0 a	0.0±0.0 a
methanol_oxidation	0.0±0.0 a	0.0±0.0 a	0.0±0.0 a	0.0±0.0 a
methylotrophy	0.0±0.0 a	0.0±0.0 a	0.0±0.0 a	0.0±0.0 a
aerobic_ammonia_oxidation	0.0±0.0 a	0.0±0.1 a	0.0±0.0 a	0.0±0.0 a
nitrification	0.0±0.0 a	0.0±0.1 a	0.0±0.0 a	0.0±0.0 a
sulfate_respiration	0.5±0.2 a	0.8±0.5 a	0.6±0.3 a	0.6±0.2 a
sulfur_respiration	0.0±0.0 a	0.0±0.0 a	0.0±0.0 a	0.0±0.0 a
respiration_of_sulfur_compounds	0.5±0.2 a	0.8±0.5 a	0.6±0.3 a	0.6±0.2 a
nitrate_denitrification	0.0±0.0 a	0.0±0.0 a	0.0±0.0 a	0.0±0.0 a
nitrite_denitrification	0.0±0.0 a	0.0±0.0 a	0.0±0.0 a	0.0±0.0 a
denitrification	0.0±0.0 a	0.0±0.0 a	0.0±0.0 a	0.0±0.0 a
chitinolysis	0.0±0.0 a	0.0±0.0 a	0.0±0.0 a	0.0±0.0 a
dark_hydrogen_oxidation	12.3±3.6 a	11.5±3.1 a	10.5±2.7 a	11.5±1.8 a
nitrogen_fixation	0.0±0.0 a	0.0±0.1 a	0.1±0.1 a	0.0±0.0 a
nitrite_respiration	0.0±0.0 a	0.0±0.0 a	0.0±0.0 a	0.0±0.0 a
cellulolysis	1.6±0.7 a	1.6±1.0 a	2.3±1.2 a	2.1±0.1 a
xylanolysis	0.1±0.0 a	0.1±0.1 ab	0.0±0.0 b	0.1±0.0 ab
dark_thiosulfate_oxidation	0.0±0.0 a	0.0±0.0 a	0.0±0.0 a	0.0±0.0 a
dark_oxidation_of_sulfur_compounds	0.0±0.0 a	0.0±0.0 a	0.0±0.0 a	0.0±0.0 a
manganese_oxidation	0.0±0.0 b	0.0±0.0 b	0.1±0.1 a	0.0±0.0 ab
manganese_respiration	0.0±0.0 a	0.0±0.0 a	0.0±0.0 a	0.0±0.0 a
fermentation	0.3±0.4 a	0.3±0.3 a	0.3±0.3 a	0.2±0.1 a
aerobic_chemoheterotrophy	27.1±5.6 a	27.2±3.6 a	28.7±2.2 a	26.7±2.0 a
invertebrate_parasites	0.1±0.0 a	0.1±0.1 a	0.1±0.0 a	0.2±0.1 a
human_pathogens_pneumonia	0.0±0.0 ab	0.0±0.1 ab	0.2±0.2 a	0.0±0.0 b
human_pathogens_all	0.3±0.3 a	0.3±0.1 a	0.5±0.2 a	0.3±0.1 a
human_gut	0.0±0.0 a	0.0±0.1 a	0.0±0.0 a	0.0±0.0 a
human_associated	0.3±0.3 a	0.3±0.1 a	0.5±0.2 a	0.3±0.1 a
mammal_gut	0.0±0.0 a	0.0±0.1 a	0.0±0.0 a	0.0±0.0 a
animal_parasites_or_symbionts	2.6±1.4 ab	2.1±0.8 b	5.6±2.3 a	4.6±0.8 a
oil_bioremediation	0.0±0.0 a	0.0±0.0 a	0.0±0.0 a	0.0±0.0 a
aromatic_hydrocarbon_degradation	0.0±0.0 a	0.0±0.0 a	0.1±0.1 a	0.1±0.0 a
aromatic_compound_degradation	0.1±0.0 a	0.1±0.1 a	0.2±0.2 a	0.1±0.0 a
aliphatic_non_methane_hydrocarbon_degradation	0.0±0.0 a	0.0±0.0 a	0.0±0.0 a	0.0±0.0 a
hydrocarbon_degradation	0.0±0.0 a	0.0±0.0 a	0.1±0.1 a	0.1±0.0 a
iron_respiration	0.4±0.1 a	0.1±0.1 b	0.4±0.3 ab	0.4±0.1 a

nitrate_respiration	0.1±0.1 ab	0.1±0.0 ab	0.2±0.1 a	0.1±0.0 b
nitrate_reduction	0.9±0.8 a	0.7±0.2 a	0.9±0.4 a	0.7±0.2 a
nitrogen_respiration	0.1±0.1 ab	0.1±0.0 ab	0.2±0.1 a	0.1±0.0 b
fumarate_respiration	0.0±0.0 a	0.0±0.0 a	0.0±0.0 a	0.0±0.0 a
intracellular_parasites	0.4±0.2 a	0.3±0.1 a	0.5±0.2 a	0.7±0.4 a
predatory_or_exoparasitic	0.1±0.2 a	0.1±0.1 a	0.1±0.0 a	0.0±0.0 a
chloroplasts	0.5±0.4 a	0.1±0.0 a	0.7±0.6 a	0.7±0.9 a
photosynthetic_cyanobacteria	0.5±0.4 a	0.1±0.0 a	0.7±0.6 a	0.7±0.9 a
anoxygenic_photoautotrophy_S_oxidizing	0.0±0.0 a	0.0±0.0 a	0.0±0.0 a	0.0±0.0 a
anoxygenic_photoautotrophy	0.0±0.0 a	0.0±0.0 a	0.0±0.0 a	0.0±0.0 a
oxygenic_photoautotrophy	0.5±0.4 a	0.1±0.0 a	0.7±0.6 a	0.7±0.9 a
photoautotrophy	0.5±0.4 a	0.1±0.0 a	0.7±0.6 a	0.7±0.9 a
aerobic_anoxygenic_phototrophy	0.0±0.0 a	0.0±0.0 a	0.0±0.0 a	0.0±0.0 a
photoheterotrophy	0.0±0.0 a	0.0±0.0 a	0.0±0.0 a	0.0±0.0 a
phototrophy	0.5±0.4 ab	0.1±0.0 b	0.7±0.5 a	0.7±0.9 ab
plastic_degradation	0.0±0.0 a	0.0±0.0 a	0.0±0.1 a	0.0±0.0 a
ureolysis	0.2±0.2 ab	0.1±0.0 b	0.2±0.0 a	0.3±0.1 a
chemoheterotrophy	30.9±3.7 a	31.7±3.7 a	31.7±2.1 a	29.5±1.3 a
anaerobic_chemoheterotrophy	3.8±2.3 a	4.4±0.8 a	3.0±1.2 a	2.8±0.8 a

3 Additional results with different rarefaction thresholds

Here we report the results of alpha (Table i) and beta diversities of fungi and bacterial communities considering the most stringent rarefaction threshold (with differences tested through Kruskal-Wallis and Dunn's tests). Thus, fungi sequences (of bulk topsoil and rhizospheres) were rarefied at 33,404. For bacteria, data were rarefied to 30,470.

Table i. Fungi and bacteria alpha diversities with fungi samples rarefied to 33,404 and bacterial samples rarefied to 30,470.

		**Fungi**			**Bacteria**		
	**Soil/Tree species**	**Phyla richness**	**ASVs Richness**	**Shannon-Wiener (ASVs)**	**Phyla richness**	**ASVs Richness**	**Shannon-Wiener (ASVs)**
2-year-old plantation	Topsoil	7.5±1.3	263.8±134	4.3±0.2	29±2.8	1790±110	6.7±0.1
	A. mangium	8.3±1.5	302.3±62.4	4±0.7	27.3±0.6	1470.3±310	6.3±0.3
	Saccharum spp.	8±1	301.3±40	4.1±0.4	31.7±0.6	2036±250	6.7±0.2
	C. nucifera	8±1	269±50	4.3±0.2	25.3±6.1	1387.3±547	6±0.8
	I. cylindrica	5.7±0.6	155.3±61	3.6±0.3	30.7±1.5	2112.7±272	6.7±0.2
10-year-old plantation	Topsoil	9.3±0.5	307±84.1	4.2±0.3	30.8±1.3	1941±302	6.6±0.2
	A. mangium	9±0	303.3±140	4.3±0.3	28.7±2.1	1758.3±166	6.4±0.1
	P. indicus	8.6±0.6	166±55	4±0.3	28.3±1.5	1569.7±37	6.5±0.03
	S. macrophylla	8±1.4‡	311.5±23‡	4±0.02‡	28.7±2.5	1839±100	6.6±0.08

Testing the beta diversity among samples of the rhizosphere and respective topsoil of the fungal and bacterial communities showed similar microbial composition, with all adjusted p-values scoring above 0.05.
